# High glucose–induced Smad3 linker phosphorylation and CCN2 expression are inhibited by dapagliflozin in a diabetic tubule epithelial cell model

**DOI:** 10.1042/BSR20203947

**Published:** 2021-06-21

**Authors:** Xinlu Pan, Mysore K. Phanish, Deborah L. Baines, Mark E.C. Dockrell

**Affiliations:** 1 South West Thames Institute for Renal Research, London, SM5 1AA, U.K.; 2 St Georges’ University of London, SW17 0RE, U.K.; 3 Epsom and St Helier Hospitals NHS Trust, SM5 1AA, U.K.

**Keywords:** Diabetic Kidney Disease, Glucose, Fibrosis, Smad3, Glucose Transporters, Dapagliflozin

## Abstract

Background: In the kidney glucose is freely filtered by the glomerulus and, mainly, reabsorbed by sodium glucose cotransporter 2 (SGLT2) expressed in the early proximal tubule. Human proximal tubule epithelial cells (PTECs) undergo pathological and fibrotic changes seen in diabetic kidney disease (DKD) in response to elevated glucose. We developed a specific *in vitro* model of DKD using primary human PTECs with exposure to high D-glucose and TGF-β1 and propose a role for SGLT2 inhibition in regulating fibrosis. Methods: Western blotting was performed to detect cellular and secreted proteins as well as phosphorylated intracellular signalling proteins. qPCR was used to detect CCN2 RNA. Gamma glutamyl transferase (GT) activity staining was performed to confirm PTEC phenotype. SGLT2 and ERK inhibition on high D-glucose, 25 mM, and TGF-β1, 0.75 ng/ml, treated cells was explored using dapagliflozin and U0126, respectively. Results**:** Only the combination of high D-glucose and TGF-β1 treatment significantly up-regulated CCN2 RNA and protein expression. This increase was significantly ameliorated by dapagliflozin. High D-glucose treatment raised phospho ERK which was also inhibited by dapagliflozin. TGF-β1 increased cellular phospho SSXS Smad3 serine 423 and 425, with and without high D-glucose. Glucose alone had no effect. Smad3 serine 204 phosphorylation was significantly raised by a combination of high D-glucose+TGF-β1; this rise was significantly reduced by both SGLT2 and MEK inhibition. Conclusions**:** We show that high D-glucose and TGF-β1 are both required for CCN2 expression. This treatment also caused Smad3 linker region phosphorylation. Both outcomes were inhibited by dapagliflozin. We have identified a novel SGLT2 -ERK mediated promotion of TGF-β1/Smad3 signalling inducing a pro-fibrotic growth factor secretion. Our data evince support for substantial renoprotective benefits of SGLT2 inhibition in the diabetic kidney.

## Introduction

Diabetic kidney disease (DKD) is a multifactorial condition and major complication of diabetes. Data indicate that between 20 and 40% of patients with diabetes develop diabetic kidney disease. The global prevalence of diabetes is predicted to rise from 450 to 600 million by 2040 [1]. With the number of patient diagnoses only expected to rise, the battle to prevent established kidney disease remains a prevalent healthcare challenge. The result of chronic exposure to raised glucose and spikes of severe hyperglycaemia results in pathological changes to the kidney including mesangial expansion, glomerular and tubular hypertrophy along with glomerulosclerosis and tubulointerstitial fibrosis [2]. The severity of tubulointerstitial fibrosis is an important determinant of progressive loss of renal function [3,4] and failure to intervene early in the unrelenting fibrotic process can lead to end stage renal disease. The identification of novel biomarkers of renal disease in diabetic patients such as cystatin C and kidney injury molecule-1, have contributed to an important shift to a ‘tubulocentric’ view on the development and progression of DKD with the proximal tubule epithelial cell (PTEC) being the major cell type involved in this perspective [5,6].

Sodium glucose transporters (SGLT2) inhibitors, also known as gliflozins, are the most recent class in a long line of anti-diabetic drugs approved by the FDA [7]. The underlying principle behind these gliflozins is that by blocking glucose entry at the PTEC via the inhibition of the transporter, glucose reabsorption is reduced, thus inducing glycosuria and decreasing plasma glucose concentrations [8,9]. Mutations in the SGLT2 gene are linked to familial renal glycosuria where abnormal glucose excretion into the urine occurs with no adverse effects on their health [10–12]. Evidence from these patients indicated that SGLT2 could be targeted to increase glucose loss in the urine without deleterious effects. Phlorizin was the first naturally occurring gliflozin to be discovered but was found to be a non-specific SGLT Inhibitor and dapagliflozin was later developed as an alternative. Dapagliflozin maintains a 1200 fold selectivity for hSGLT2 (*K*_i_ = 6 nM) over hSGLT1 (*K*_i_ = 360 nM) coupled with a dissociation rate that is approximately 10-fold lower [13].

Additionally, a type 2 diabetic *db/db* mouse study found that treatment with the SGLT2 inhibitor empagliflozin also reduced tubulointersitial fibrosis, observed through the significant decrease of α1(I) collagen and fibronectin abundance in kidney sections [14]. Thus, we hypothesised that reducing PTEC glucose uptake with the SGLT2 inhibitor dapagliflozin would result in a reduction in the fibrotic response in human cells. In addition to a chronic difficulty regulating circulating glucose patients with diabetes can suffer from rapid spikes in blood glucose levels which may not be reflected in HbA1c levels. We developed a model using primary human PTEC with acute exposure to high glucose to mimic these spikes.

The current diagnostic criteria for DKD include established diabetes and albuminuria. A consequence of PTEC exposure to albumin is the megalin independent expression of TGF-β1 [15]. TGF-β1 is a pleiotropic protein involved in a plethora of functions including fibrosis. One of the major molecular pathways driving renal fibrosis is believed to be the canonical TGF-β1/Smad3 pathway. Results published by Zorena et al. indicated that serum TGF-β1 levels was the most ‘discriminate power’ when compared with several other variables including blood pressure, HbA1c and creatinine in predicting diabetic outcomes in a group of ‘juvenile patients’. They determined the cut-off threshold concentration for TGF-β1 where it relates to diabetes induced complications was 0.443 ng/ml. We have therefore incorporated a threshold concentration of TGF-β1 as part of our *in vitro* model [16].

Connective tissue growth factor (CCN2), a secreted 36/38kDa pro-fibrotic marker, has also been implicated in DKD [17]. It has a characteristic doublet consistent with partial glycosylation and is known to act downstream of TGF-β1 [18]. FG-3019, a fully human monoclonal neutralising antibody against CCN2, has been shown to reduce fibrosis and significantly reduce albumin creatinine ratio in diabetic patients, supporting the proposal that CCN2 is an effective target for the treatment of DKD [19]. A clinical study showed that the abundance of urinary CCN2 N-terminal fragments increased 10-fold when comparing microalbuminuric and normoalbuminuric diabetic patients, directly correlating with the severity of proteinuria and therefore the rate of DKD progression [20].

In this work, we investigate the induction of CCN2 in primary human PTECs and have sought to determine the molecular basis underlying the pro-fibrotic response. We have delineated a role for SGLT2 in the regulation of a pro-fibrotic number of intracellular signalling cascades and hence, offer a novel target for the treatment of tubulointerstitial fibrosis in DKD.

## Materials and methods

### Cell culture

In house human primary PTECs were previously isolated and characterised by Percoll density gradient centrifugation [21], while PTECs isolated from other donors were purchased from Lonza, U.S.A. Phenotype was confirmed by Gamma GT activity (Supplementary Figure S4). Adult human dermal fibroblasts were purchased from Cell Biologics, U.S.A. In house PTECs were cultured in a mixture of Dulbecco’s Modified Eagle medium (DMEM) and Ham’s Nutrient (F12). This was supplemented with heat inactivated fetal bovine serum (FBS, 10%), insulin (5 µg/ml), transferrin (5 µg/ml), sodium selenite (5 ng/ml), hydrocortisone (36 ng/ml), epidermal growth factor (10 ng/ml), penicillin (100 U/ml), streptomycin (100 µg/ml), tri-iodothyronine (4 pg/ml) and L-glutamine (2 mM). Lonza PTECS were cultured in renal epithelial basal and growth medium (REBM™/REGM™, Lonza). Fibroblasts were cultured in complete fibroblast medium (Cell Biologics). All medium were sterile filtered using a 0.2 µM Puradisc™ (Whatman) and changed on alternate days. All cells were grown under 5% CO_2_ humidity at 37°C on T75 tissue culture flasks and 35 mm culture dishes (Corning).

### Collagen IV coating

Human placenta collagen IV (Sigma) was reconstituted in sterile 0.25% acetic acid and coated onto both tissue culture flasks and dishes (Corning) at 5 μg/cm^2^. After all culture-ware with collagen IV was exposed to UV radiation for 20 min, the coating was left to incubate overnight at room temperature. Flasks and dishes were then rinsed once using sterile PBS and air dried before use.

### Cell subculture and cryopreservation

After cells reached approximately 85% in confluence, the culture medium was removed and cell dissociated with trypsin-EDTA solution for 5 min at 37°C. When detached, the cell suspension was aspirated and centrifuged at 1500 rpm (327 ***g***) for 6 min. The supernatant was discarded and the pellet re-suspended in fresh medium. Live cells were counted after mixing with trypan blue solution (Sigma) using an automated cell counter (Bio-Rad). PTECs and fibroblasts were seeded onto 35 mm collagen IV coated dishes at a density of 2500 and 5000 cells/cm^2^, respectively.

### Treatment and inhibitors

Cells were serum starved for 24 h prior to treatment. All subsequent conditions were supplemented with 0.1% BSA. Cells were exposed to three different conditions: (i) 7 mM D-glucose (control); (ii) 7 mM D-glucose + 18 mM D-glucose (high D-glucose or D-Glu); and (iii) 7 mM D-glucose + 18 mM L- glucose (osmotic control or L-Glu), with or without TGF-β1 0.75 ng/ml (Sigma-Aldrich). We used L-glucose to account for the osmotic stress the cells are subjected to upon >7 mM glucose treatment as osmotic stress is known to activate ERK in renal epithelial cells [22]. The SGLT2 inhibitor dapagliflozin (Cayman Chemical) was administered at 0.1, 1, or 10 nM while MEK/ERK inhibitor U0126 (Sigma-Aldrich) was administered at 10 μM. Treatment times ranged from 5 min to 24 h.

### Protein lysis and assay

The medium was removed from cell cultures and stored at −70°C. Cells were then rinsed with ice cold PBS. To obtain whole cell protein, cells were scraped from dishes and homogenised in ice cold lysis buffer (Tris-HCl, 20 mM at pH 7.2, EDTA, 1 mM, SDS 0.1%, sodium deoxycholate 0.5%, Triton X-100 1%) and 10 ml/l EASYpack protease inhibitor and PHOSstop phosphatase inhibitor cocktails (Roche). This mixture was placed on ice for 15 min prior to centrifugation for 10 min at 10000 rpm (6720 ***g***). The lysed protein in the supernatant was then removed and stored at −70°C until use. Bicinchoninic acid assay (Pierce) was used to determine protein concentration of the lysate as per the manufacturer’s protocol.

### Western blot

**Figure 1:**
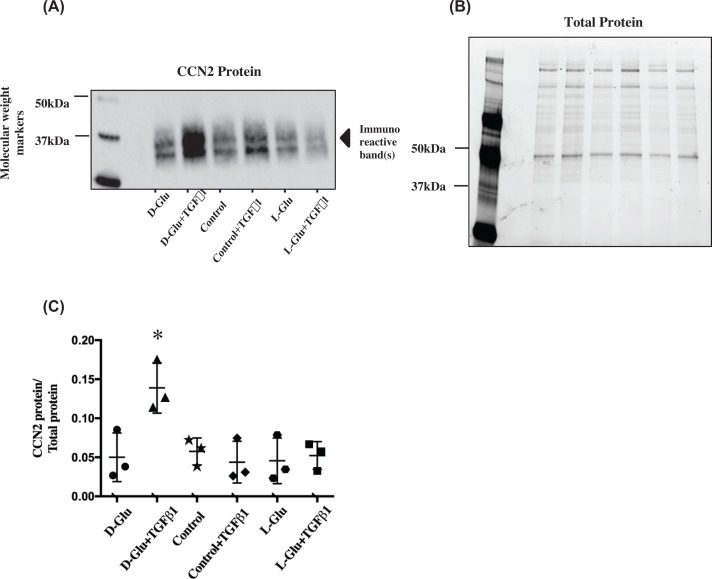
Effects of high glucose and TGF-β1 stimulation on PTECs that express CCN2 (**A** and** B**) Image of Western blot immunostained for secreted CCN2 protein in PTECs under various treatments and the ‘Mini-PROTEAN TGX Stain-Free’ gel used for normalisation purposes. (**C**) Data points for secreted CCN2 (∼36/38 kDa) abundance normalised for total protein. The Mini-PROTEAN TGX stain-free gels used for normalisation contain a trihalocompound that automatically produces fluorescence when covalently crosslinked to protein tryptophan residues. After the proteins are separated by electrophoresis, crosslinking is carried out by exposing the gel to UV light. This is the band density normalisation process used for all subsequent experiments. D-Glu+TGF-β1 treatment significantly up-regulated CCN2 secretion. *P<0.05 compared with all other treatments, *n*=3. Black circles ● = D-Glu, black triangles ▲ = D-Glu+TGF-β1, black stars ★ = control, black diamonds ♦ = control + TGF-β1, black hexagons 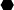 = L-Glu, black squares ▓ = L-Glu + TGF-β1. Error bars indicate SD. Quantification of all proteins in these figures were a result of the light generated by the detection antibody indexed to total protein loaded in each sample. Control = 7 mM D-glucose, Control + TGF-β1 = 7 mM D-glucose + 0.75 ng/ml TGF-β1, D-Glu = 7 mM D-glucose + 18 mM D-glucose, L-Glu = 7 mM D-glucose + 18 mM L-glucose, D-Glu + TGF-β1 = 7 mM D-glucose + 18 mM D-glucose + 0.75 ng/ml TGF-β1, L-Glu + TGF-β1 = 7 mM D-glucose + 18 mM L-glucose + 0.75 ng/ml TGF-β1. This legend key is applicable to all subsequent figures.

Equal amounts of protein (20/25 µg per sample) were boiled in 2× Laemmli buffer (Bio-Rad) with 5% mercaptoethanol for 5 min at 95°C and separated on either 4-15%, 7.5% or 10% ‘Mini-PROTEAN TGX Stain-Free’ gels (Bio-Rad) for ∼25 min at 220 V. Images of gel proteins were captured on the ChemiDoc™ Touch system (Bio-Rad) before the transfer onto polyvinylidene (PVDF) membranes for total protein normalisation and adjustment for loading. Blots were blocked with 5% non-fat milk, in tris-buffered saline-tween (TBST), and incubated overnight at 4°C with rabbit polyclonal anti SGLT2 (1:500, Santa Cruz), rabbit polyclonal anti CCN2 (1:500, Invitrogen), rabbit polyclonal anti-phospho-ERK (1:1000, CST), rabbit polyclonal anti phospho-Smad3(SS^p^XS^p^) (1:1000, CST), or rabbit polyclonal anti phospho-Smad3 LR serine 204 (1:1000, Abcam). Blots were washed in TBST three times and incubated with an anti-rabbit IgG horseradish peroxidase (HRP) conjugate secondary antibody for an hour at room temperature (1:2000, New England Biolabs). Immunostained proteins were also captured on the ChemiDoc™ imager. Protein abundance analysis was carried out using ImageLab™ software (Bio-Rad) where target protein density was expressed relative to the amount of total protein. Polyacrylamide gel containing a proprietary trihalo compound to make proteins fluorescent directly in the gel with a short photoactivation, allowing the immediate visualisation of proteins. Following polyacrylamide gel elxtropheresis. Gels were placed in a ChemiDoc™ Imaging (Bio-Rad) system for activation by exposure to UV light for 1 min. A stain-free image was taken using the imaging system for total protein measurement in each lane. Image data were analysed using Image 4.1 software (Bio-Rad). Stain-Free gels provide a linear dynamic range between 10 and 80 µg of total protein load. The total density for each lane is measured from the blot and a lane profile is obtained. The background is adjusted in such a way that the total background is subtracted from the sum of density of all the bands in each lane (referred to as the rolling disk background subtraction algorithm).The software interprets the raw data in three dimensions with the length and width of the band defined by the ‘Lanes and Bands’ tool in concert with the ‘Lane Profile’ tool such that the chemiluminescent signal emitted from the blot is registered in the third dimension as a peak rising out of the blot surface. The density of a given band was measured as the total volume under the three-dimensional peak, which could be viewed in two dimensions using the ‘Lane Profile’ tool to adjust the precise width of the band to account for the area under the peak of interest. An example total protein gel is presented in Figure 1.

### Heparin bead purification

For the analysis of proteins with heparin binding domains, media from treated cells were mixed together at a 10:1 ratio with heparin-agarose bead suspension (Sigma-Aldrich). Medium and bead suspension were mixed on a roller for 6 h. Tubes were then centrifuged at 4°C for 10 min at 10,000 rpm (10,060 ***g***) (Hettich Mikro), and the supernatant was discarded. The beads were then rinsed in ice-cold PBS twice and heparin bound proteins were subsequently extracted and separated as detailed in the above Western blot section.

### qPCR

Complementary DNA (cDNA) was produced via a reverse transcription polymerase chain reaction (RT-PCR) using the High Capacity cDNA Reverse Transcription kit (Life Technologies). Sample cDNA, glyceraldehyde 3-phosphate dehydrogenase (GAPDH) housekeeping primers, CCN2 Taqman gene expression assay and Taqman gene expression master mix were then mixed together on a 96-well PCR plate (Bio-Rad). Real time quantification of gene target(s) was carried out using the CFX1000 Real Time system thermocycler (Bio-Rad) under the pre-optimised qPCR thermal cycler program. CFX manager software was used to analyse the results. The fold changes in cDNA expression were presented as values normalised to the housekeeping gene using the ∆*C*t method.

The following primer probe sets (Life Technologies) were all pre-validated at standardised conditions:

**Table:** 

Gene	Assay ID	Reference sequence
GAPDH	Hs99999905_m1	NM_002046.3
CCN2/CTGF	Hs00170014_m1	NM_001901.2

### Gamma GT staining

Confluent dishes of PTECs were stained with a solution of 6 mM glutamic acid γ-(4-methoxy-β-naphthylamide), 4 mM glycyl-glycine, 1.1 mM Fast Blue B Salt, 100 mM NaCl, and 25 mM Tris at pH 7.4 (Sigma Aldrich) in deionised water for 45 min at room temperature. Cells were then washed with PBS before examination under the microscope. Distinct orange-red staining confirmed presence of the enzyme.

### Statistical analysis

Statistical analysis on all treatments was performed on GraphPad Prism 7 (GraphPad Software Inc) where *P* values < 0.05 were considered statistically significant. Comparison between two means was carried out using unpaired *t*-test. Comparison of the means of more than two groups was carried out using analysis of variance (ANOVA) with Bonferroni’s *post hoc* test. Values are reported as mean ± standard deviation (SD).

The use of all human tissue was carried out with the approval of the South London and Surrey Research Ethics Committee, REC reference number 08/H0806/8.

## Results

### The effect of high glucose and TGF-β1 on CCN2 production in human PTECs

Upon treatment, neither exposure to TGF-β1 0.75 ng/ml nor 25 mM D-Glucose (D-Glu) was found to be sufficient to produce a significant increase in CCN2 secretion within 24 h. However, the combination of D-Glu + TGF-β1 significantly increased the secretion of the glycosylated and non-glycosylated isoforms of CCN2, 38 and 36 kDa (*P*<0.05, *n*=3) (Figure 1A–C).

### The effect of SGLT2 inhibition on CCN2 protein secretion and mRNA expression

TGF-β1 (0.75 ng/ml) induction of CCN2 was dependent on high D-glucose; this was seen in both glycosylated and non-glycosylated protein, suggesting a transcriptional or post-translational regulation.

**Figure 2:**
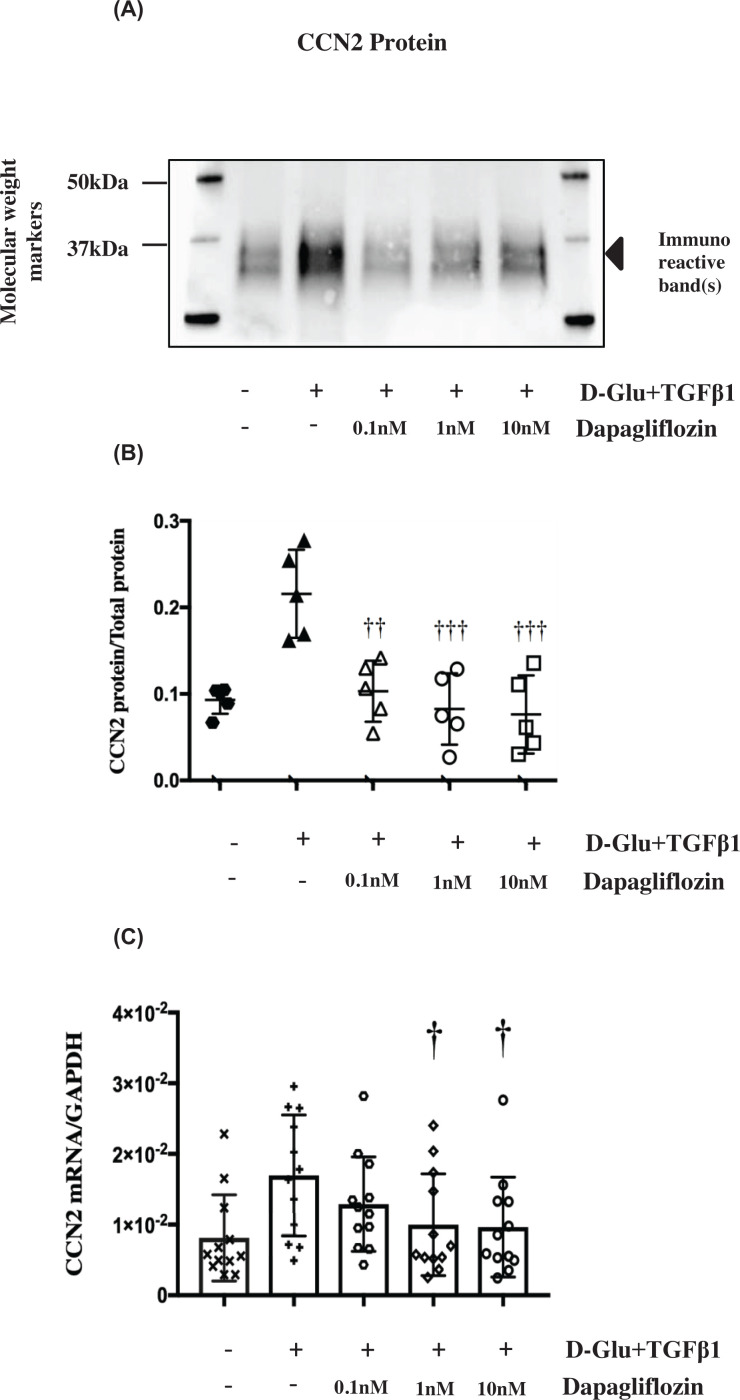
Effects of Dapagliflozin (dapa) on up-regulated CCN2 mRNA and protein in PTECs (**A** and** B**) Image of Western blot immunostained for secreted CCN2 protein in PTECs under various treatments. (**C**) Data points for secreted CCN2 (36–38 kDa) abundance normalised for total protein as mentioned above in Figure 1. Dapagliflozin at all three concentrations significantly attenuated CCN2 protein down to control levels; ††*P*<0.01 compared with D-Glu + TGF-β1, †††*P*<0.001 compared with D-Glu+TGF-β1, *n*=5. Black hexagons 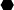 = control, upward black triangles ▲ = D-Glu+TGF-β1, white triangles Δ = D-Glu+TGF-β1+0.1 nM dapa, white circles ○ = D-Glu+TGF-β1+1 nM dapa, white squares □ = D-Glu+TGF-β1+ 10 nM dapa. (**D**) Effects of 0.1, 1 and 10 nM dapagliflozin on up-regulated CCN2 mRNA (Δ*C*t of CCN2 to GAPDH) in PTECs. Dapagliflozin at 1 and 10 nM significantly attenuated CCN2 relative mRNA abundance to control levels. †*P*<0.05 compared with D-Glu+TGF-β1. Black X **X** = control, black cross **+** = D-Glu+TGF-β1, white hexagons 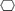 = D-Glu+TGF-β1+0.1 nM dapa, white diamonds ◊ = D-Glu+TGF-β1+1 nM dapa, white circles ○ = D-glu+TGF-β1+ 10 nM dapa, *n*=12. Error bars indicate SD. Quantification of all proteins in these figures were a result of the light generated by the detection antibody indexed to total protein loaded in each sample. Control = 7 mM D-glucose, Control + TGF-β1 = 7 mM D-glucose + 0.75 ng/ml TGF-β1, D-Glu = 7 mM D-glucose + 18 mM D-glucose, L-Glu = 7 mM D-glucose + 18 mM L-glucose, D-Glu+TGF-β1 = 7 mM D-glucose + 18 mM D-glucose + 0.75 ng/ml TGF-β1, L-Glu + TGF-β1 = 7 mM D-glucose + 18 mM L-glucose + 0.75 ng/ml TGF-β1. This legend key is applicable to all subsequent figures.

The effect of SGLT2 inhibition using dapagliflozin was assessed at the level of RNA and protein expression. Dapagliflozin (0.1–10 nM) completely inhibited D-Glu+TGF-β1 induced CCN2 protein secretion to levels comparable with the L-glucose control (*P*<0.01, *n*=5) (Figure 2A,B), CCN2 mRNA abundance was significantly reduced by the two higher concentrations (1 and 10 nM) at 24 h (*P*<0.05, *n*=12) (Figure 2C). The effect of D-Glu+TGF-β1 with and without dapagliflozin was observed in cells from all donors (Supplementary Figure S2).

### Investigating the point of convergence of high glucose and TGF-β1 stimulation

We next investigated putative signalling pathways that may be involved in facilitating this synergistic up-regulation of CCN2. Based on our previous work [22], we investigated the role of ERK and Smad3. In addition to the canonical phosphorylation of the MH2 domain by the TGFβ receptor complex, Smad3 activity can be regulated by phosphorylation of the linker region [23]. Although ERK and Smad3 are both established downstream mediators of TGF-β signalling, the linker region of Smad3 has been studied to a much lesser extent.

**Figure 3:**
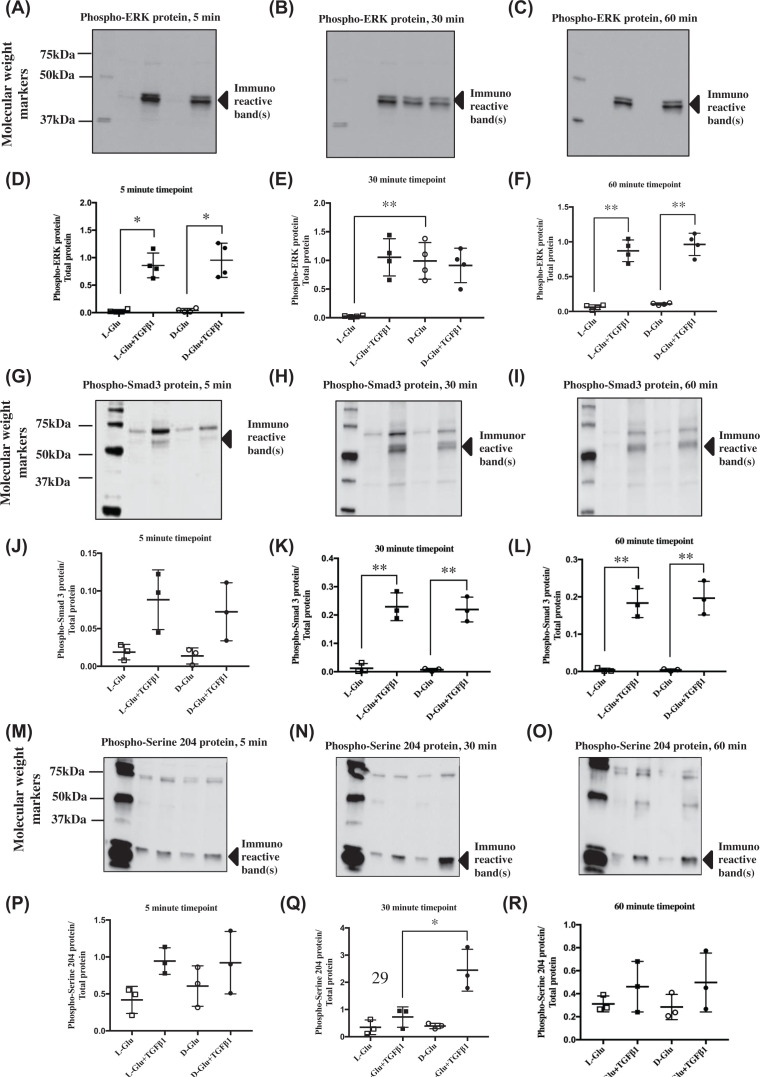
Phosphorylation state of various signalling pathways upon treatment with high D-glucose and TGF-β1 in PTECs (**A–C**) Image of western blots immunostained for cellular phospho-ERK protein in PTECs under various treatments and time points. (**D–F**) Data points for cellular phospho-ERK (∼40 kDa) abundance normalised for total protein as mentioned above in Figure 1. TGF-β1 induced ERK phosphorylation significantly, +/- high D-glucose for 60 min, while exclusive high D-glucose treatment phosphorylated ERK at 30 min; *P<0.05. (**G–I**) Image of Western blots immunostained for cellular phospho-Smad3 protein in PTECs under various treatments and time points; (G and H) Smad3 phosphorylated at serine 423/425; (I and J) Smad3 phosphorylated at serine 204. (**J–L**) Data points for cellular phospho-Smad3 (∼50 kDa) abundance normalised for total protein. TGF-β1 significantly phosphorylated Smad3, +/- high D-glucose from 15 min onwards; **P<0.01. (**M–O**) Image of Western blots immunostained for cellular phospho-serine 204 of Smad3 LR protein in PTECs under various treatments and time points. (**P–R**) Data points for cellular phospho-serine 204 of Smad3 LR protein (∼25 kDa) abundance normalised for total protein. D-Glu+TGF-β1 treatment at 30 min significantly phosphorylated serine 204 compared to L-Glu+TGF-β1 at 30 min; *P<0.05, *n*=3–4. Black squares ▓ = L-Glu+TGF-β1, black circles ● = D-Glu+TGF-β1, white circles ○ = D-Glu, white squares □ = L-Glu. Error bars indicate SD. Quantification of all phosphorylated proteins in these figures were a result of the light generated by the detection antibody indexed to total protein loaded in each sample. Control = 7 mM D-glucose, Control + TGF-β1 = 7 mM D-glucose + 0.75 ng/ml TGF-β1, D-Glu = 7 mM D-glucose + 18 mM D-glucose, L-Glu = 7 mM D-glucose + 18 mM L-glucose, D-Glu + TGF-β1 = 7 mM D-glucose + 18 mM D-glucose + 0.75 ng/ml TGF-β1, L-Glu + TGF-β1 = 7 mM D-glucose + 18 mM L-glucose + 0.75 ng/ml TGF-β1. This legend key is applicable to all subsequent figures.

There was no detectable change in phosphorylated ERK levels over 5-60 min in the presence of the osmotic control (L-Glu). Treatment with TGF-β1 phosphorylated ERK from 5 to 60 min whilst high D-glucose alone increased phosphorylated ERK in an acute manner at 30 min before it returned to basal levels by 60 min (*P*<0.05, *n*=4) (Figure 3A–F).

Similar to ERK, Smad3 was rapidly phosphorylated at the MH2 domain (serine 423 and 425) by TGF-β1, which reached statistical significance at 30 min. (*P*<0.01, *n*=3) (Figure 3G–L).

Phosphorylated Smad3 LR (Linker Region) serine 204 was detected with immunoreactive bands of approximately 25, 50 and 75 kDa visible. There was marked induction of Smad3 LR serine 204 phosphorylation by the specific combination of D-Glu+TGF-β1 at 30 min compared with all other treatments (*P*<0.05, *n*=3).

### The effect of MEK and SGLT2 inhibition on phosphorylation of Smad3 serine 204 and ERK

**Figure 4:**
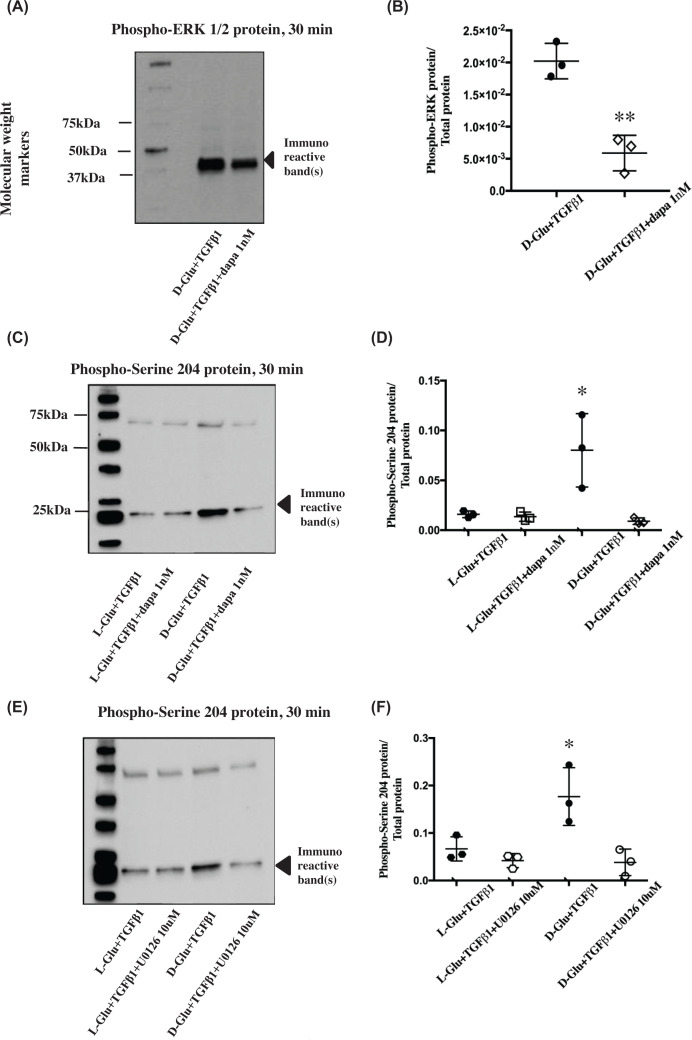
Phosphorylation state of Smad3 serine 204 and ERK after U0126 and dapagliflozin treatment (**A**) Image of Western blot immunostained for cellular phospho-ERK (∼40 kDa) protein in PTECs treated with dapagliflozin and (**B**) the corresponding data points for cellular phospho-ERK abundance normalised for total protein as mentioned above in Figure 1. Dapagliflozin significantly reduced the cellular content of phosphorylated ERK (∼40 kDa). **P<0.01 compared with D-Glu+TGF-β1+dapa 1 nM, *n*=3. (**C**) Image of Western blot immunostained for cellular phospho-serine 204 of Smad3 LR protein in PTECs treated with dapagliflozin and (**D**) the corresponding data points for cellular phospho-serine 204 of Smad3 LR abundance normalised for total protein. Dapagliflozin (1 nM) significantly reduced the cellular content of phosphorylated serine 204. *P<0.05 compared with D-Glu + TGF-β1 + dapa 1 nM, *n*=3. (**E**) Image of Western blots immunostained for cellular phospho-serine 204 of Smad3 LR protein in PTECs treated with U0126 and (**F**) the corresponding data points for cellular phospho-serine 204 of Smad3 LR (∼25kDa) abundance normalised for total protein. U0126 (10 μM) significantly reduced the cellular content of Smad3 phosphorylated serine 204 at 30 min. **P<0.05 compared to D-Glu + TGF-β1 + U0126, *n*=3. Black hexagons 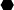 = L-Glu + TGF-β1, white hexagons 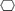 = L-Glu + TGF-β1 + U0126, black circles ● = D-Glu + TGF-β1, white circles ○ = D-Glu + TGF-β1 + U0126, white squares □ = L-Glu + TGF-β1, white diamonds ◊ = D-Glu + TGF-β1 + dapa 1 nM. Quantification of all phosphorylated proteins in these figures were a result of the light generated by the detection antibody indexed to total protein loaded in each sample.

As shown above, high glucose treatment resulted in elevated phosphorylated ERK from 15 to 45 min. Co-incubation of Dapagliflozin with D-Glu+TGFβ1 significantly inhibited ERK phosphorylation observed following D-Glu + TGFβ1 treatment (Figure 4 A,B) (*P*<0.01, *n*=3). As previously observed, phosphorylation of Smad3 at serine 204 was significantly higher in the PTECS treated with high glucose compared with the osmotic control in the presence of TGF-β1. Dapagliflozin treatment was also able to significantly reduce Smad3 serine 204 phosphorylation (Figure 4C,D) (*P*<0.05, *n*=3). Finally, to test whether ERK phosphorylation is upstream of Smad3 serine 204 phosphorylation U0126, a MEK inhibitor was used (U0126 efficacy in reducing ERK phosphorylation was confirmed in Supplementary Figure S3). Smad3 serine 204 phosphorylation was significantly reduced to basal levels by pre-incubation of cells with 10 μM U0126 (*P*<0.05, *n*=3; Figure 4E,F).

**Figure 5:**
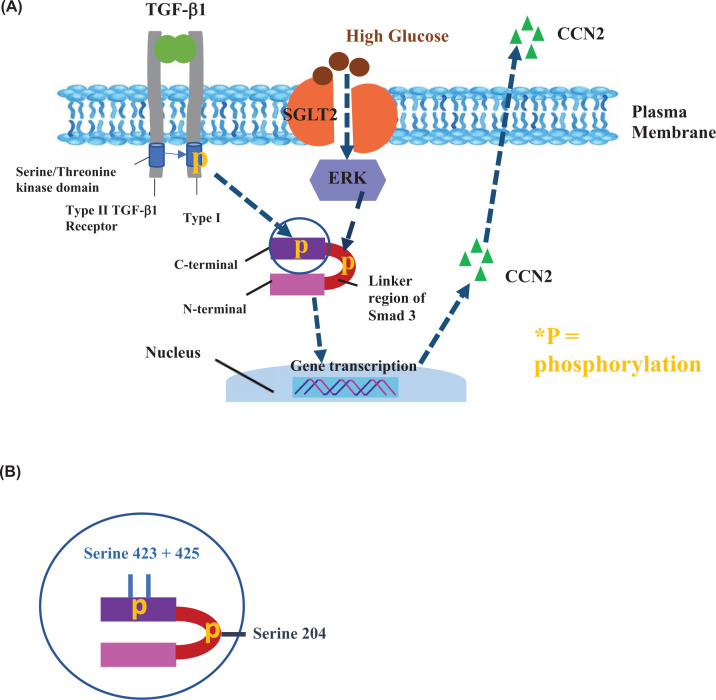
Schematic of proposed signalling mechanism: glucose mediated pro-fibrotic CCN2 induction (**A**) Typically, the TGF-β1 type 2 receptor phosphorylates the cytoplasmic domain of the type 1 receptor, which then proceeds to phosphorylate Smad3 on the MH2 domain, (**B**) specifically at serine 423 and 425. The fully activated Smad complex is then translocated to the nucleus where it controls gene transcription. Under hyperglycaemic conditions, ERK is also activated and phosphorylates the serine 204 located on the linker region between the C (SSXS) and N terminal domain. This then increases TGF-β specific signalling of the complex, thereby potentiating transcriptional activity at the nucleus. Control = 7 mM D-glucose, Control + TGF-β1 = 7 mM D-glucose + 0.75 ng/ml TGF-β1, D-Glu = 7 mM D-glucose + 18 mM D-glucose, L-Glu = 7 mM D-glucose + 18 mM L-glucose, D-Glu + TGF-β1 = 7 mM D-glucose + 18 mM D-glucose + 0.75 ng/ml TGF-β1, L-Glu + TGF-β1 = 7 mM D-glucose + 18 mM L-glucose + 0.75 ng/ml TGF-β1. This legend key is applicable to all subsequent figures.

## Discussion

We have studied the role of extracellular D-glucose in mediating TGF-β1/Smad3 signalling and modulating the expression of CCN2 in human proximal tubule cells. The CCN2 promoter contains a Smad-binding element (SBE) that is a well-documented target of TGFβ1/Smad3. There are two apical glucose transporters expressed in the proximal tubule: SGLT1 and SGLT2. SGLT2 is a high capacity/low affinity glucose transporter primarily localised in the S1 and S2 segment of the proximal tubule whilst SGLT1 is a low capacity, high affinity glucose transporter found in the S3 segment. Our study specifically investigated SGLT2 using dapagliflozin which has an approximate 1200-fold selectivity for SGLT2 over SGLT1 [13]. To activate TGFβ1/Smad3, we incubated cells with 0.75 ng/ml TGF-β1 which resulted in significant phosphorylation of Smad 3 MH2 SSVS domain. This domain is the target of the TGFβ type 1 receptor (TβRI), however, at this dose, this was insufficient to induce CCN2 expression. Co-incubation of TGFβ1 with high D-glucose significantly induced CCN2 expression. This was shown to be SGLT2 dependent. We then go on to describe the dapagliflozin/U0126 sensitive glucose induction of serine 204 phosphorylation on a truncated isoform of Smad3.

The primary PTECs used in these experiments were from three separate donors and contain cells derived from all three segments. Therefore, we expect that our results may differ from those using transformed cells lines as the latter are derived from a single clonal expansion. They were cultured on collagen IV, the main extracellular matrix component in the tubular basement membrane (TBM) of the human kidney [24]. This allows for a similar TBM cellular interaction as seen in cells *in vivo* which is important for the regulation of the cell phenotype. Our primary cultures of PTECs expressed both SGLT1 and 2 mRNA. SGLT2 protein was also consistently detected by Western blotting of approximately 25 μg protein (Supplementary Figure S4) with kidney cortical lysate providing positive control for the detection of SGLT2 protein. Fibroblasts were also run alongside PTECs to act as a negative control (Supplementary Figure S1).

We chose to normalise our target band densities against the total protein visualised in our electrophoresis gel using the trademarked stain-free imaging technology recently developed by Biorad. As briefly mentioned in our results section, this technology employs a patented trihalocompound in a polyacrylamide gel which strengthens the fluorescent signal of tryptophan amino acids after a short photo-activation upon exposure to UV light. As these fluorophores are covalently bound to the protein, they can be easily captured without the need for a laborious staining and de-staining protocol. We opted to use this method instead of the traditional housekeeping protein normalisation, which is often unreliable, as it allows researchers to acquire more accurate data by dividing band densities with total lane protein.

Our study has investigated the efficacy of the diabetic class of drugs gliflozins on ameliorating diabetes associated fibrotic changes in human renal cells by targeting the underlying molecular mechanisms. There is currently no pharmacotherapy for renal fibrosis. We have focused on dapagliflozin, which was licensed for use in the US in October 2019 after the DECLARE-TIMI 58 trial showed that the drug successfully reduced the risk of hospitalisation for heart failure (HF) in adults with type 2 diabetes [25]. The data we have presented is derived from a cell culture model of DKD. Cells were exposed to raised glucose levels, to replicate hyperglycaemic peaks seen in patients, and TGF-β1. Albuminuria is a common characteristic of DKD and albumin stimulates TGF-β1 expression in PTECs. There is also evidence for significantly elevated urinary TGF-β1 in patients with diabetes when compared with healthy controls [26]. We believe this model recreates many important features of diabetic tubular disease observed in patients and can be a valuable tool for the study of some aspects of DKD, particularly the role of SGLT2.

TGF-β1 is a pleiotropic protein involved in a plethora of crucial functions including cell differentiation, wound repair and inflammation [27,28]. Smads are the canonical intracellular signalling molecules activated by TGF-β1. Following binding of TGF-β1 the type II receptor recruits and phosphorylates the type I receptor. The type I receptor in turn recruits receptor Smad dimers and phosphorylates the SSXS motif at the carboxy-terminal. The phosphorylated receptor Smads can form heteromeric complexes with Smad4. This heterotrimeric complex can then translocate to the nucleus to regulate transcription. In parallel, noncanonical signalling acts through Smad independent protein kinases such as MEK/ERK and p38 [29]. TGF-β1 is a key driver of fibrosis in the kidney and has been proposed as a target for chronic kidney disease since 1998 [30]. TGF-β1/Smad3 signalling has been suggested as the main molecular mechanism in renal fibrosis due to several studies with various models [31]. However, targeting TGF-β in renal disease has proved difficult [32]. Alternative therapies, particularly those using drugs already licensed for use are attractive options.

CCN2 is a modular matricellular protein that acts downstream of TGFβ1 [33]. We observed a clear and significant increase in CCN2 mediated by SGLT2 when cells were exposed to a combination of TGF-β1 and elevated glucose (25 mM) for 24 h. SGLT2 involvement was deduced from the experiment using dapagliflozin. There is a discrepancy in the effect of dapagliflozin between protein and mRNA, 0.1 nM only significantly inhibited protein expression. We would postulate that this is due to the fact that CCN2 is an early immediate gene with transient RNA expression and the lowest concentration of dapagliflozin used by us flattens the curve but does not result in a significant effect at 24 h.

Work from ourselves and others have previously shown that TGF- β1 is able to induce CCN2 on its own, after 12–24 h in primary PTECs and other cell types only when the concentration is 5 ng/ml or higher [22,34]. This concentration of TGF-β1 also induces epithelial–mesenchymal transition (EMT) in breast cancer cells and renal PTEC [35,36]. There are no published reports of PTEC EMT in human DKD. In the present study, we did not observe any loss of phenotype after the cells were subjected to a combination of a lower concentration of TGF-β1 in the presence of hyperglycaemia, suggesting PTECs retained their epithelial phenotype at the time of CCN2 production and secretion. The effects we observed were dependent on both glucose concentration and TGF-β1, which provides a possible reason why DKD only occurs in a minority of patients with diabetes and strengthens the argument that fibrosis may depend on a two-hit model. In contrast, a research group recently found that high glucose (25 mM) treatment alone was sufficient enough to induce a significant accumulation of fibronectin and collagen IV protein, commonly observed during tubulointerstitial fibrosis [37]. However, it is worth noting that they did not use a primary cell line from multiple donors but instead utilised a transformed HK-2 cell line which is less archetypal of the *in vivo* situation. In addition, Wu and Derynk [38] previously published a glucose induced Smad3 C terminal phosphorylation, not observed in the work presented above. This may also be due to the difference in the cell model as they utilised murine cells grown on plastic culture dishes.

We found that hyperglycaemia alone and in combination with TGFβ1 increased phosphorylated ERK, consistent with findings from Fujita et al. Their results also showed that LLC-PK1 proximal tubule cell hypertrophy induced by high glucose was subdued with the ERK inhibitor PD98059 [39]. A separate group found that ERK inhibition by both U0126 and PD98059 reduced the CCN2 expression and fibronectin accumulation that was originally up-regulated in hyperglycaemic vascular smooth muscle cells [40]. Hayashida et al. [41] also discovered that high glucose (20 mM) activated extracellular regulated signalling kinase (ERK) and, in the presence of TGF- β1, elicited a rise in α1(I) collagen promoter activity and gene expression. This glucose-stimulated increase in promoter activity was ameliorated by the MEK inhibitor PD98059. Our work demonstrates that glucose induced ERK phosphorylation is SGLT2 dependent. Interestingly, in a study of colon cancer cells Saito et al. found dapagliflozin administration increased the cellular content of phosphorylated ERK. However, the concentration used from this group was 0.25 μM [42]. The IC50 for dapagliflozin inhibition of SGLT2 is reported to be approximately 1 nM [43], and the authors concluded that the effect observed was likely to be independent of SGLT2 inhibition.

In our experiments the presence of 25mM glucose with TGFβ1 induced a cascade of signalling proteins and gene expression that was sensitive to SGLT2 inhibition. Glucose entry through SGLT2 is accompanied by equimolar sodium entry, hence increased glucose entry is accompanied by increased sodium entry. PTEC sodium levels are closely controlled by a number of apical and basolateral sodium channels; SGLT is the only glucose channel in PTEC. Hence, it is unlikely that the effects observed by us are a result of raised intracellular sodium. Furthermore, glucose-induced ERK has been observed in cells that do not express sodium/glucose co-transport, as described above [41,44]. Never the less the effect of intracellular sodium on Smad signalling remains undefined and may merit enquiry.

Our results demonstrated TGF-β1, 0.75 ng/ml, significantly increased Smad3 linker phosphorylation in the presence of raised glucose. Results previously published by our group showed that TGF-β1 induced CCN2 secretion is dependent on Smad3 through the selective use of Smad3 small interfering RNA [45]. This suggests that the dapagliflozin sensitive ERK mediated phosphorylation of Smad3 Serine 204 may be the critical point of convergence of TGF-β1 and glucose in our experiments, however we have not yet demonstrated its direct involvement in the activation of CCN2. The Hayashida/Schnaper group reported that blocking ERK in renal mesangial cells also managed to inhibit both the phosphorylation of linker region serine 204 and Smad3 mediated collagen 1A2 promoter activation. The constructs they created that specifically lacked serine 204 also reduced Smad3 α2(I) collagen promoter activity, further indicating a key role for it in the development of renal fibrosis [23]. In the same cells, TGF-β1 was able to phosphorylate the serine within the linker region of Smad3, and this was similarly reversed upon MEK inhibition [46]. Taken together these data strongly support the case that targeting SGLT2 could also inhibit the glucose-TGF-β1/ERK/Smad3 signalling cascade and subsequent key elements of renal fibrosis in our PTECs.

Interestingly, it appears from the results presented here that glucose alone and in combination with TGF-β1 causes phosphorylation of a truncated isoform (∼25kDa) of Smad3 which is dimerised with a full length Smad3 (∼50 kDa) phosphorylated at the SSXS domain. This would result in a product of approximately 75kDa, as observed on our cellular phospho-Smad3 and cellular phospho-serine 204 blots in Figure 3. We have not yet fully characterised the structure of this isoform and this 25 kDa band is not consistent with current reports in literature, which usually indicate a full length ∼50 kDa size. It was only detectable by antibodies targeting between amino acids 200 and 432, suggesting that this truncated form lacks the MH1 domain. Typically, the N-terminal MH1 domain of Smad3 is responsible for DNA binding via the CAGAC Smad-binding element (SBE) [47]. However, another type of DNA sequence identified as a Smad-binding motif are G/C rich regions. There is evidence in literature that Smad3 is also able to bind to these GC motifs that lack CAGAC sequences [48]. Smad3 forms homo and hetero trimers, with Smad4. Thus we hypothesise that our particular model involves full-sized receptor phosphorylated Smad3 binding with the truncated Smad3 (that lacks the MH1 domain) and forming a heterotrimer with Smad4 before translocating to the nucleus to regulate gene transcription. This ‘R-Smads heterotrimer’ model was previously depicted by Macias et al [49]. We propose a novel TGFβ/Smad3 signalling pathway where a dimer consisting of a full length Smad3 and a truncated form is phosphorylated on the former at the C-terminal SSXS at receptor level and later by ERK on the linker region. This proposed pathway merits further investigation.

In summary, we have identified a mechanism for an SGLT2 mediated fibrogenic pathway in a diabetic milieu and a cellular signalling cascade mediating the pro-fibrotic effect. We propose that high glucose acts on or via SGLT2 to activate MEK/ERK signalling while TGF-β1 is simultaneously activating its own receptor. These two events converge on different phosphorylation sites on Smad3 leading to up-regulation of pro-fibrotic protein CCN2. Our results provide molecular basis for improvements in renal outcomes demonstrated in patients treated with an SGLT2 inhibitor. Inhibition of SGLT2 may provide a novel therapeutic approach for the treatment of DKD by limiting tubular interstitial fibrosis.

## Supplementary Material

Supplementary Figures S1-S4

## Data Availability

The datasets/resources generated during and/or analysed for the current study are available from the corresponding author upon reasonable request.

## Abbreviations

cDNA: complementary DNA
DKD: diabetic kidney disease
DMEM: Dulbecco’s modified eagle medium
ERK: extracellular regulated kinase
FBS: foetal bovine serum
GAPDH: glyceraldehyde 3-phosphate dehydrogenase (GAPDH)
GT: glutamyl transferase
HRP: horseradish peroxidase
LR: linker region
PTEC: proximal tubule epithelial cell
qPCR: quantitative polymerase chain reaction
RT-PCR: reverse transcription polymerase chain reaction
SGLT2: sodium glucose cotransporter 2
TBST: Tris-buffered saline tween
TGF-β1: transforming growth factor beta 1
