# Supplementary material for: High glucose–induced Smad3 linker phosphorylation and CCN2 expression are inhibited by dapagliflozin in a diabetic tubule epithelial cell model
Source: Biosci Rep. 2021 Jun 21;41(6):BSR20203947. doi: 10.1042/BSR20203947 (PMC8220447; doi:10.1042/BSR20203947)
Supplement: Supplementary Figures S1-S4 [file BSR-2020-3947_supp.pdf]

Figure 1A uncropped blot

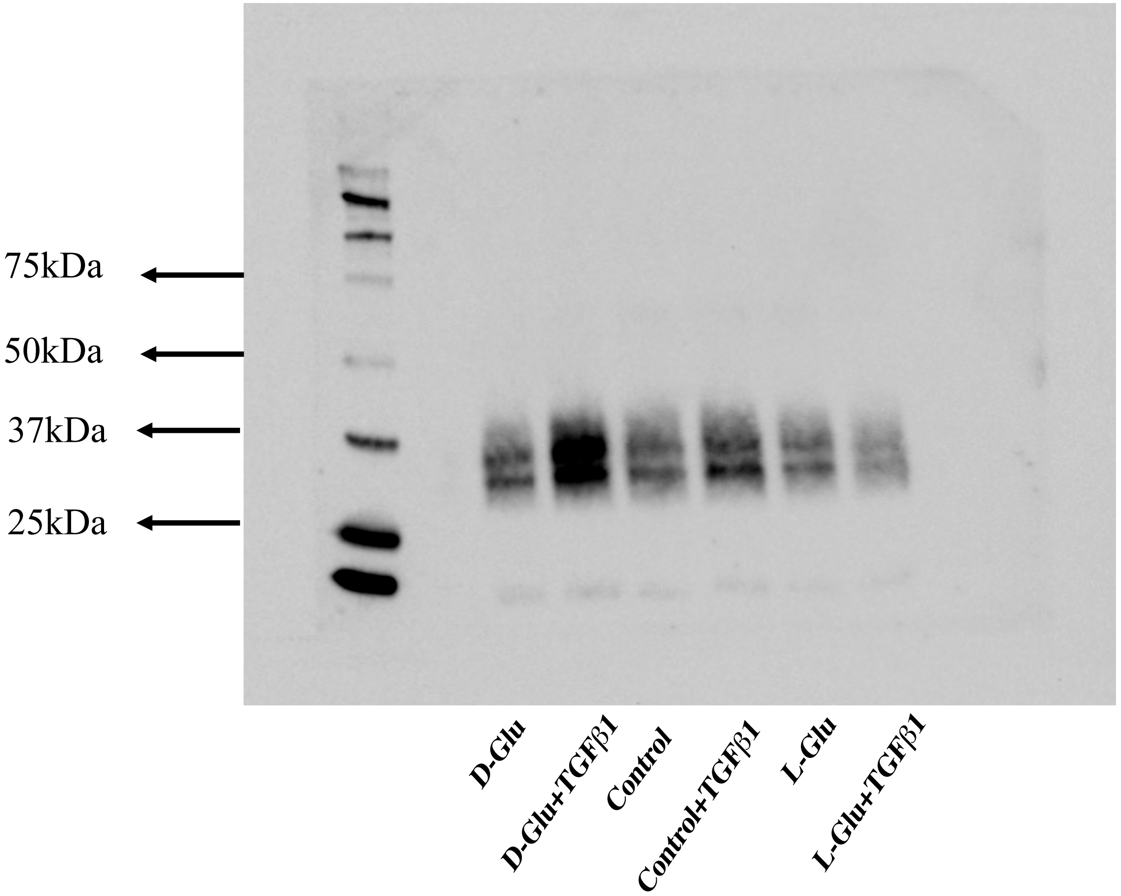

S. Figure 1
uncropped blot

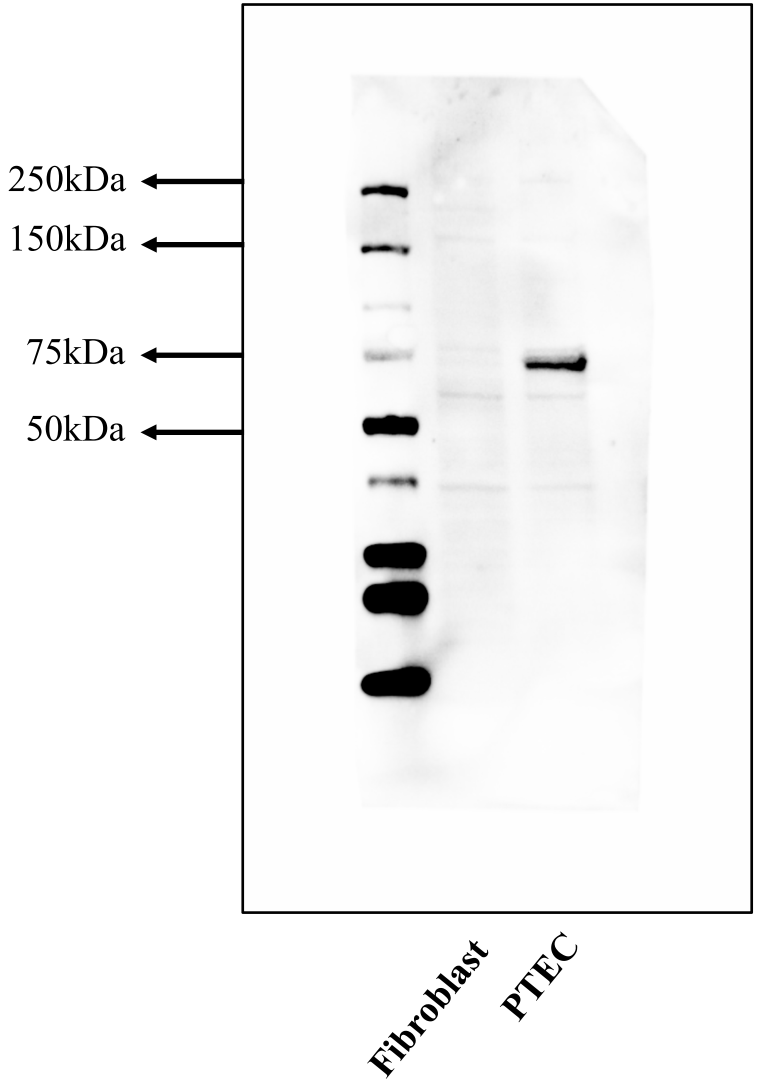

Supplemental Figure 1: Representative SGLT2 western blot image after running basal human primary PTECS alongside a negative control.

There was no expression of SGLT2 expression in the fibroblasts under our specific conditions.

Figure 2A
uncropped blot

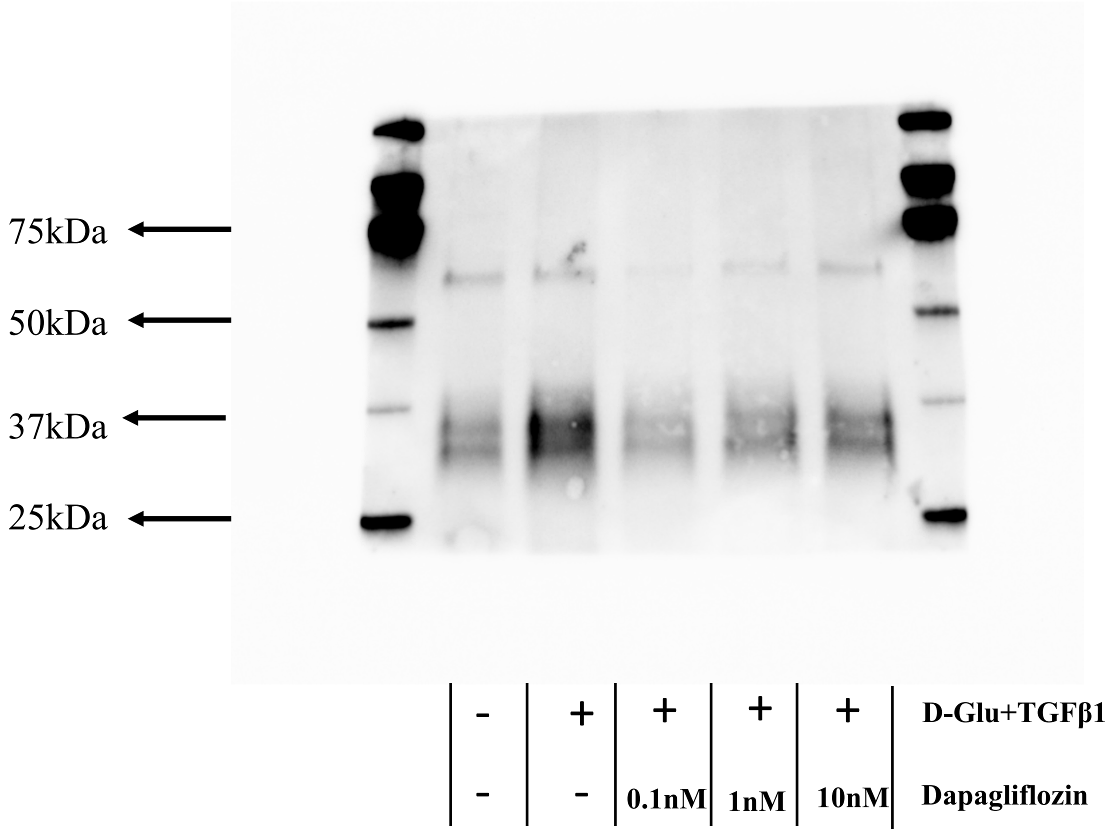

Supplemental Figure 2: Individual donor responses to Dapagliflozin (dapa) treatment.

As expected, each donor had a slight variation in response to the drug, but the overall response trend remained the same.

Figure 3A
uncropped blot

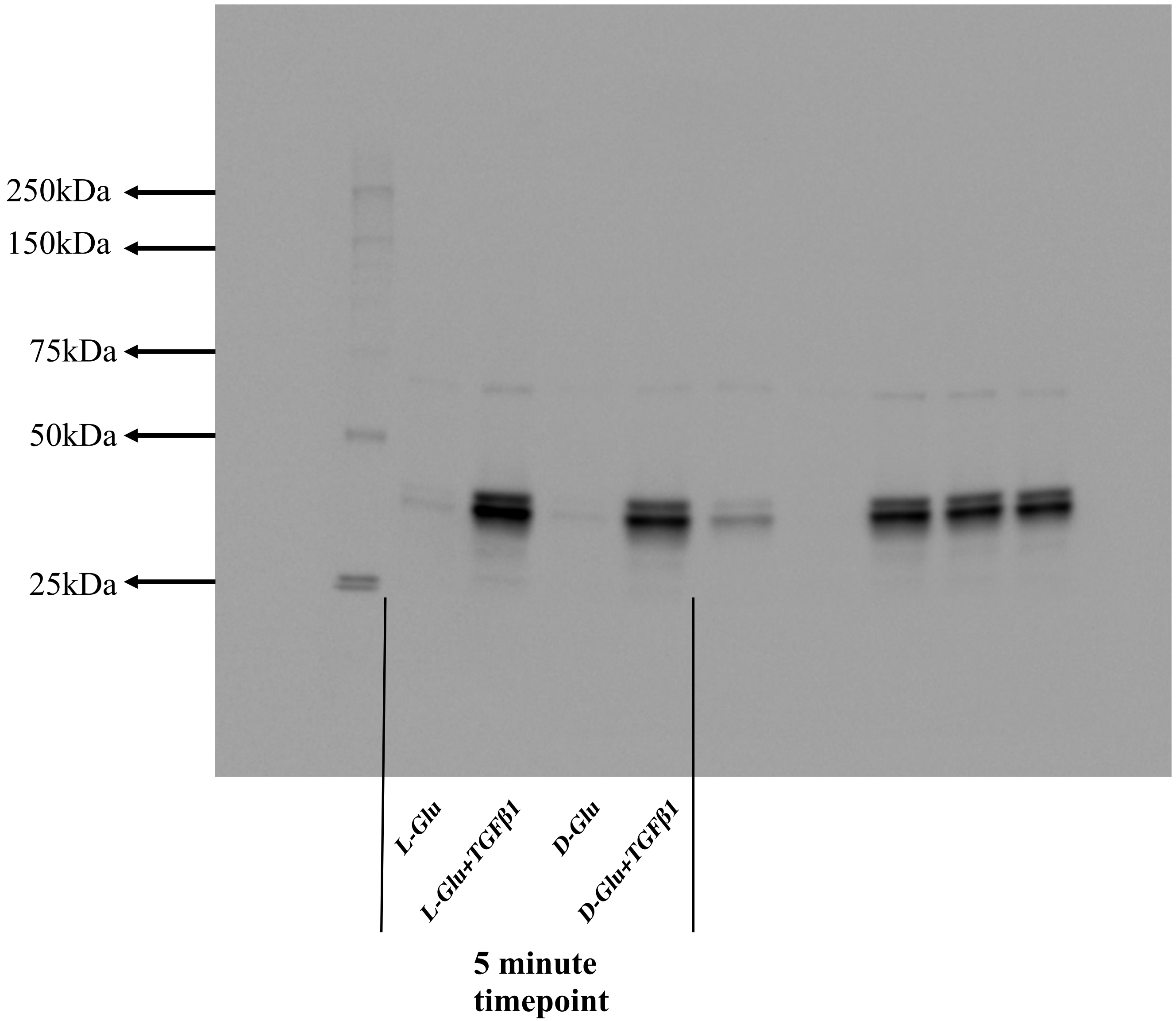

Figure 3B
uncropped blot

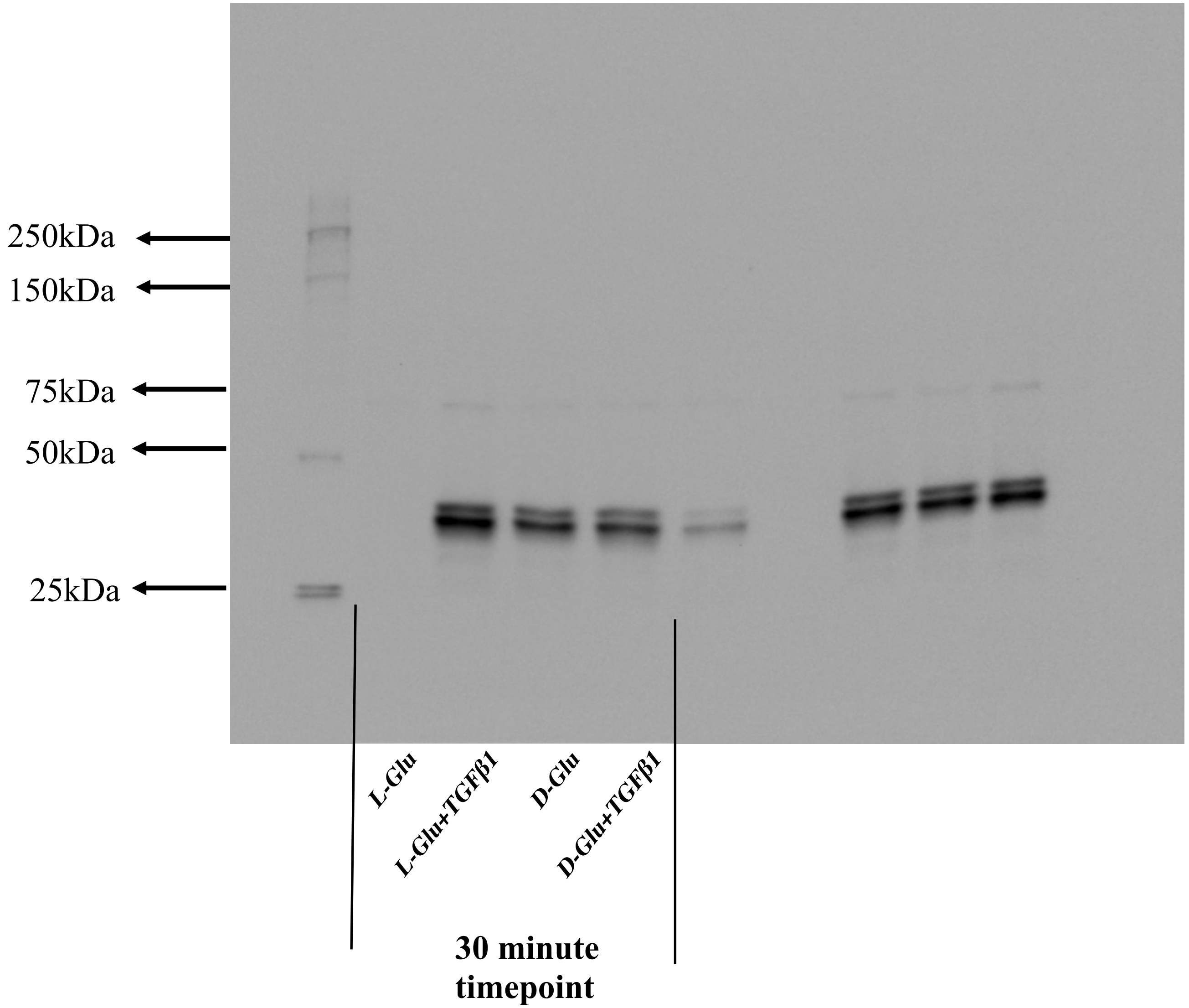

Figure 3C
uncropped blot

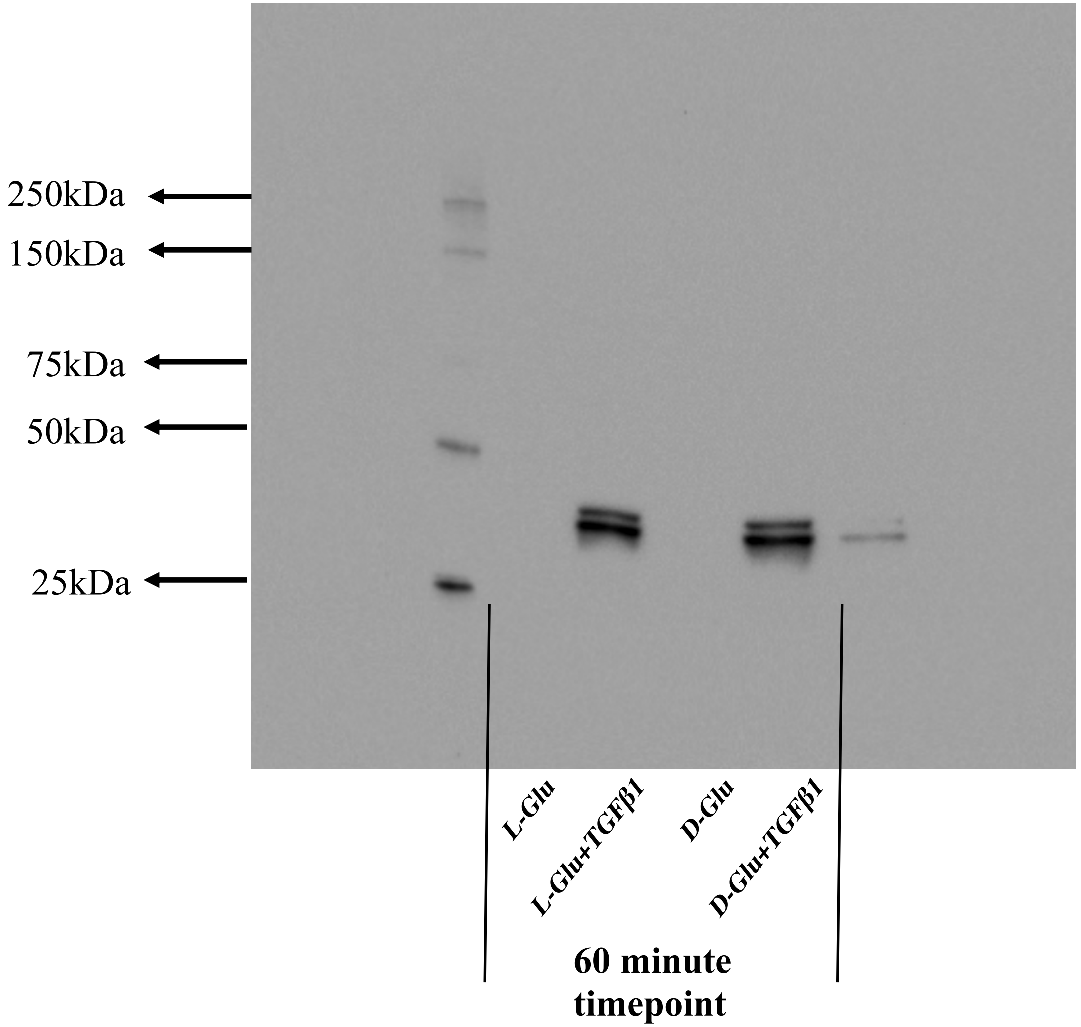

Figure 3G uncropped blot

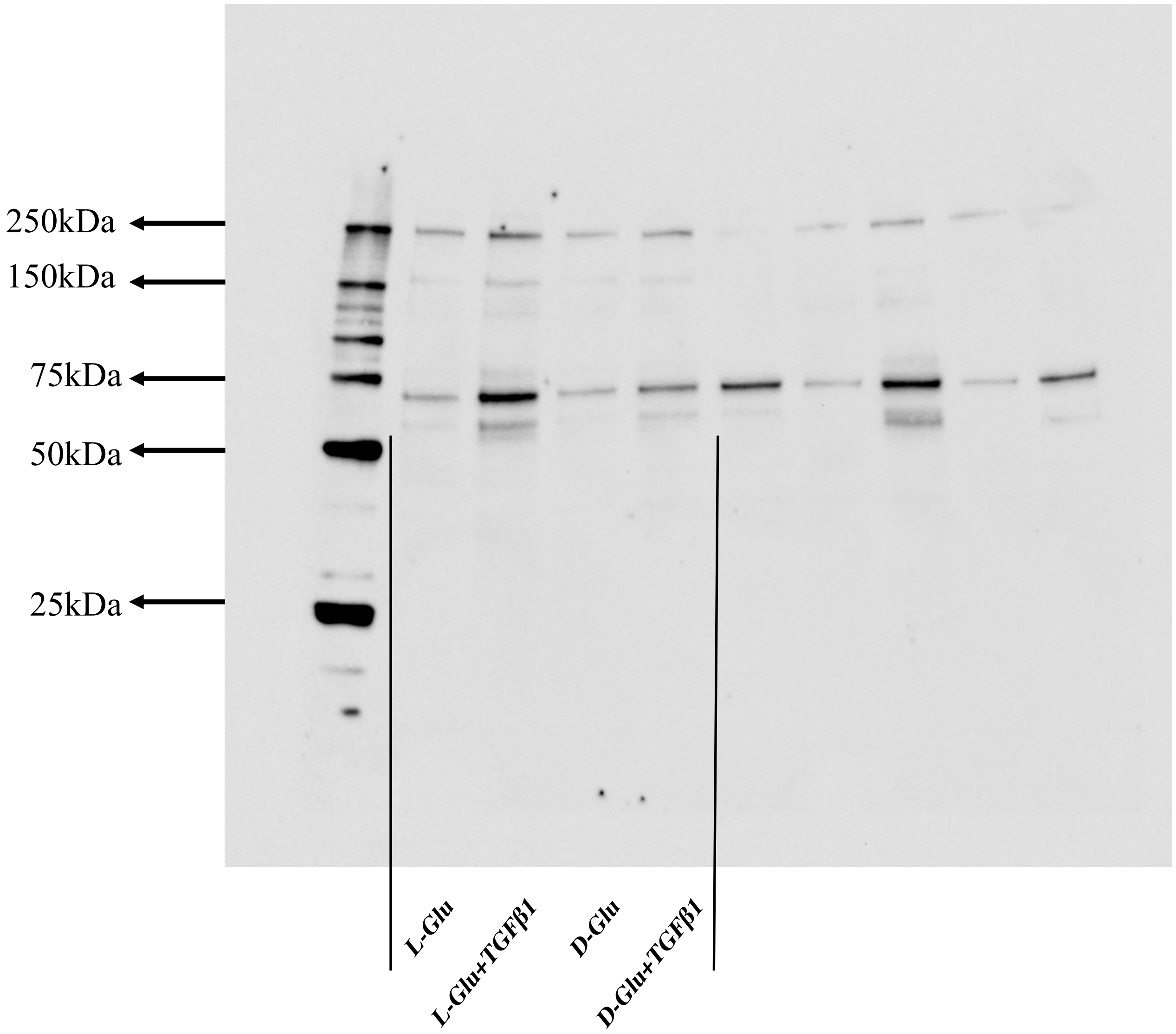

5 minute timepoint

Figure 3H
uncropped blot

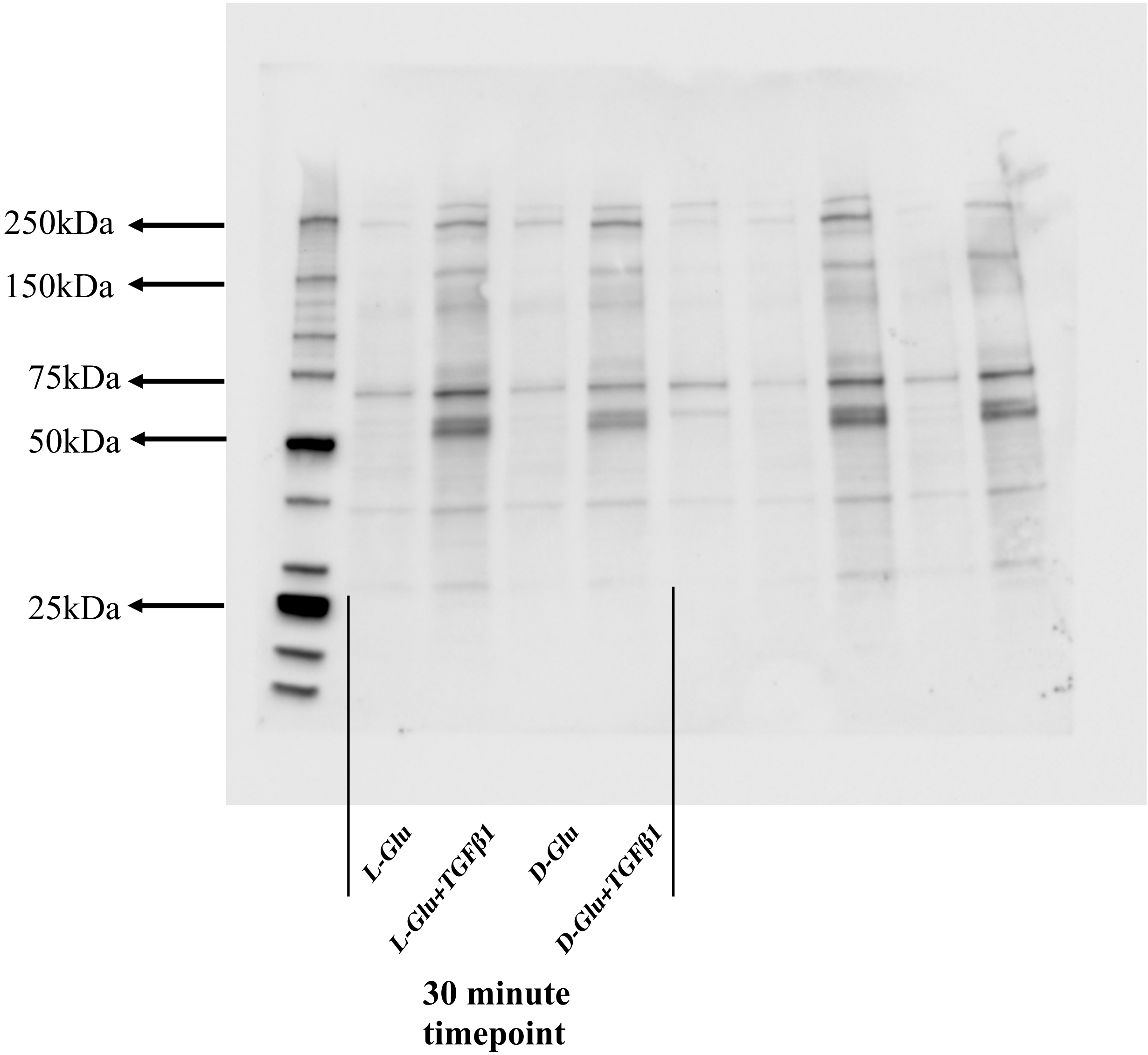

Figure 3I
uncropped blot

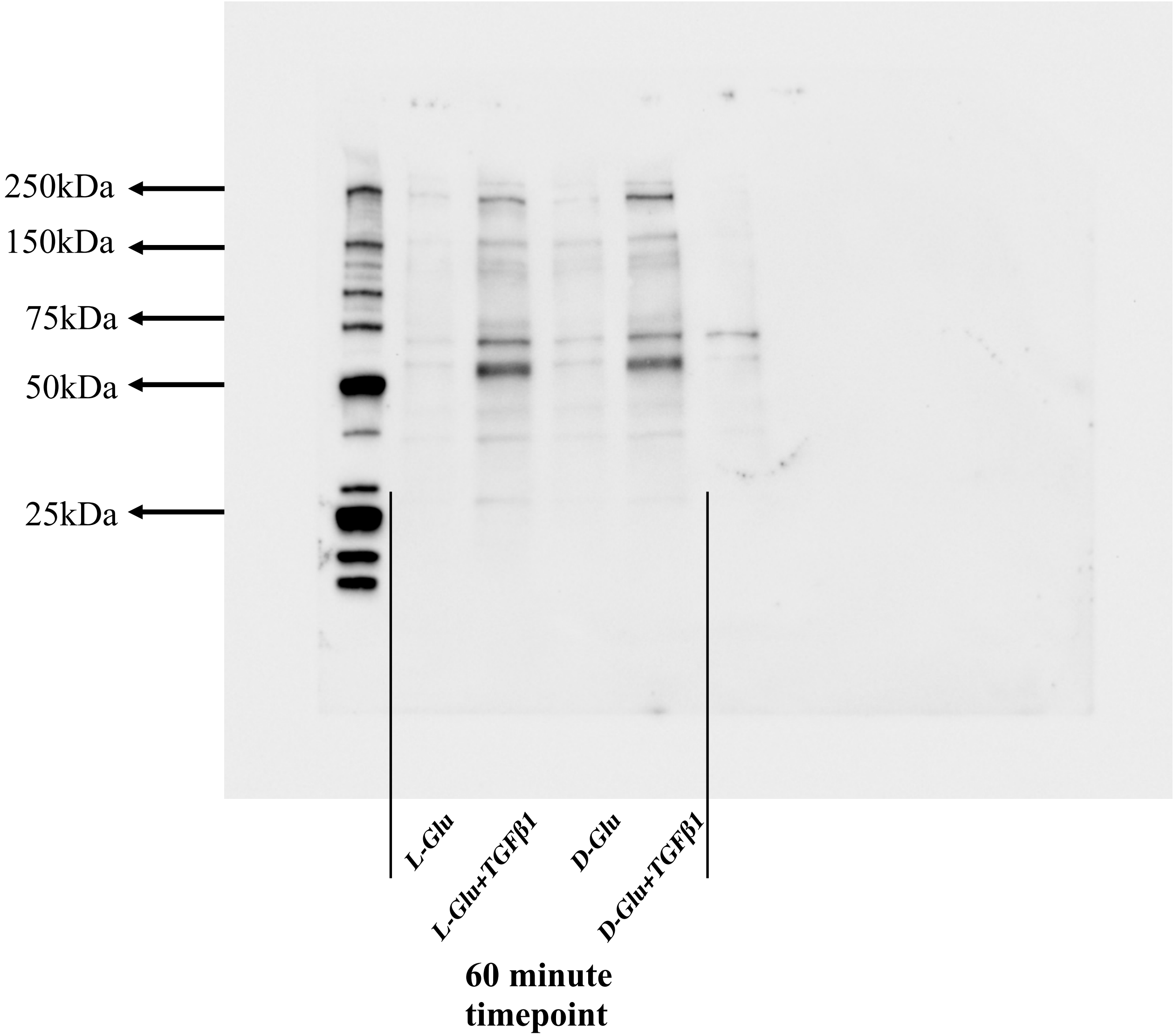

Figure 3M
uncropped blot

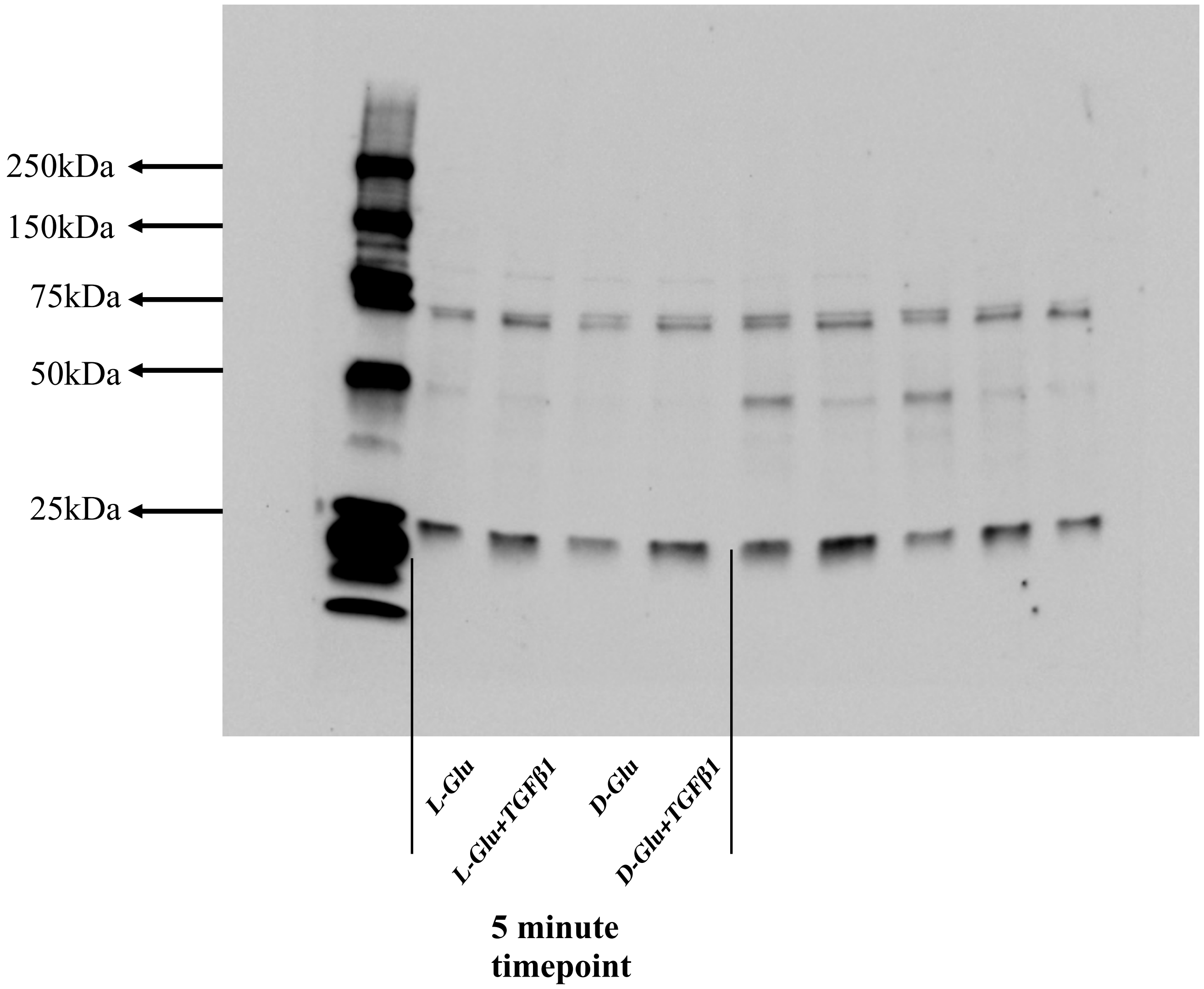

Figure 3N
uncropped blot

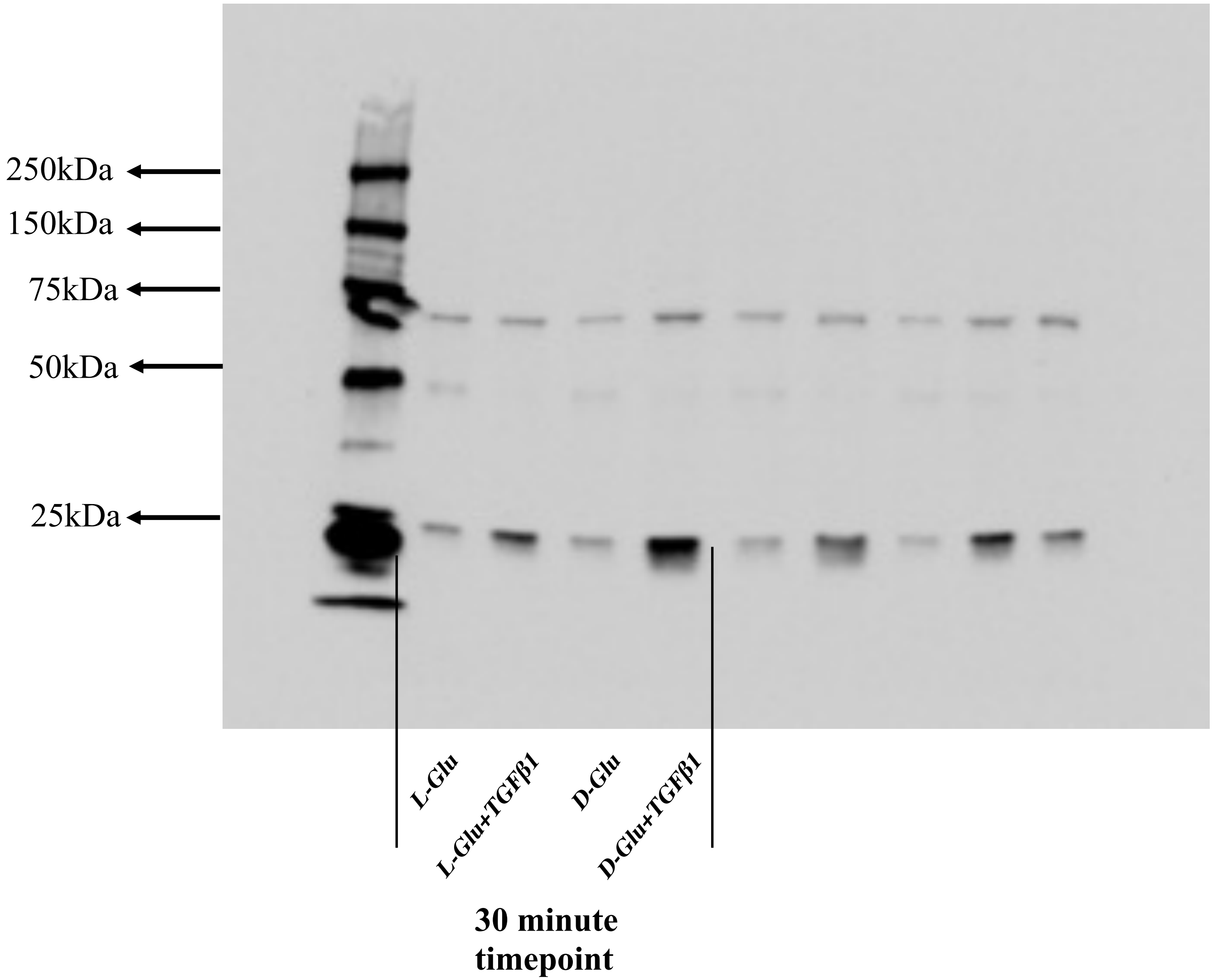

Figure 3O
uncropped blot

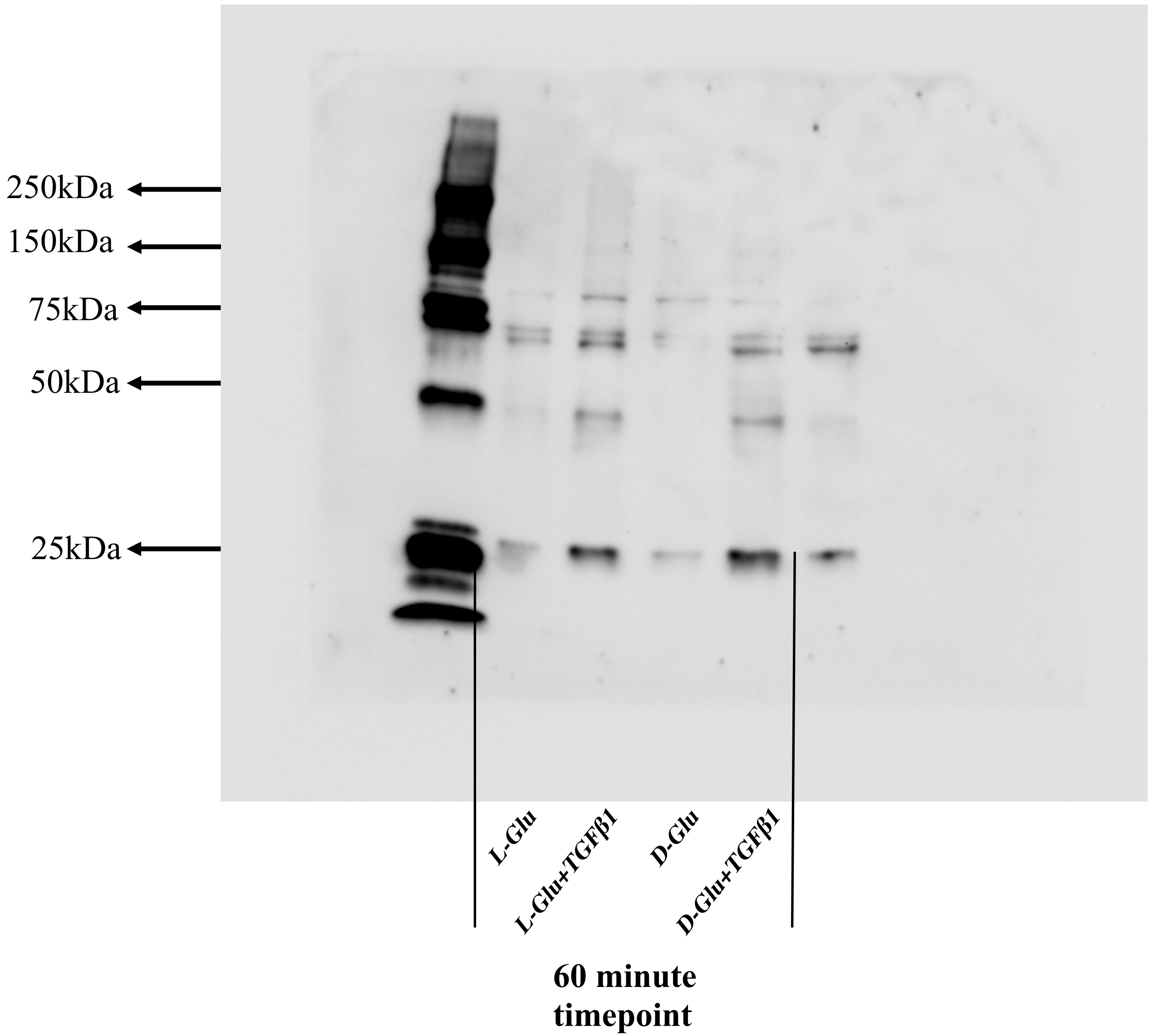

S. Figure 3
uncropped blot

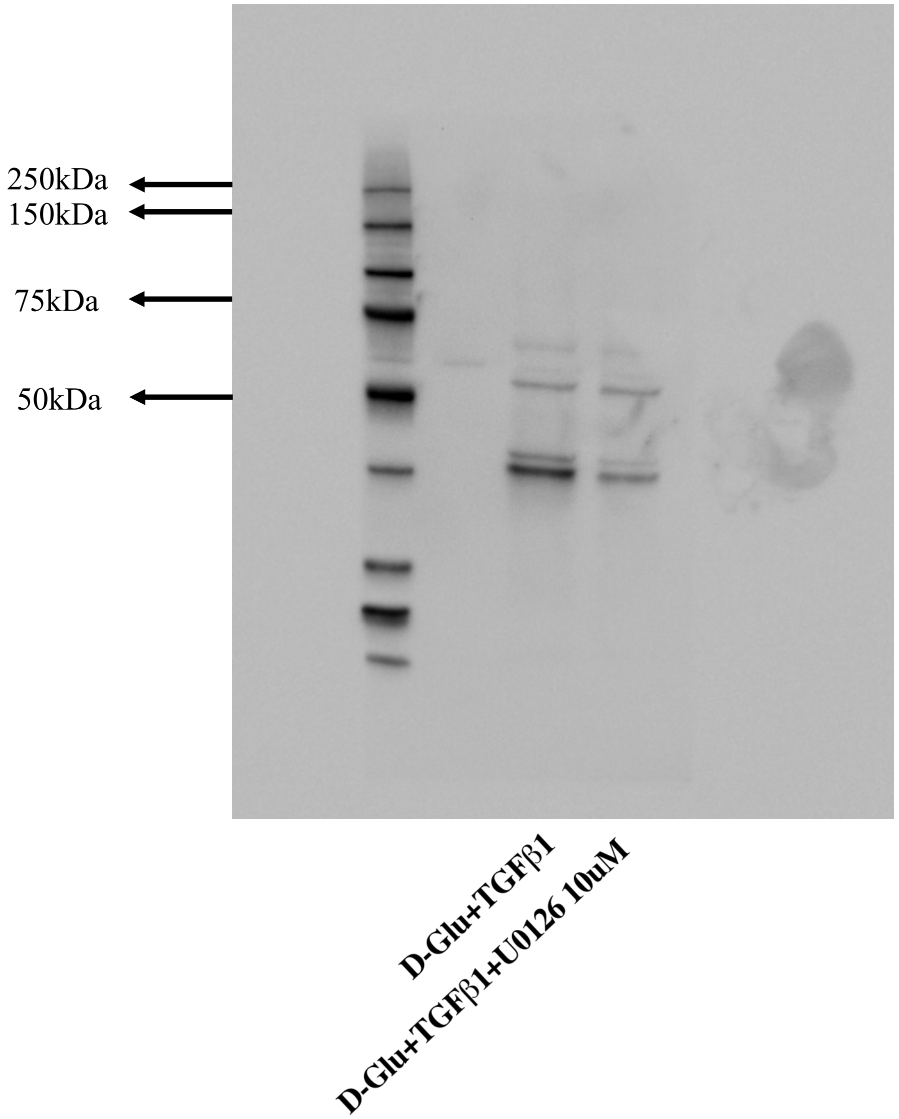

Supplemental Figure 3: Representative Phospho-ERK western blot after running PTECs treated with high glucose and TGFβ1, with/without U0126 MEK inhibitor at 10μM.
Confirmation of significant phospho-ERK knockdown by U0126 MEK inhibitor.

Figure 4A
uncropped blot

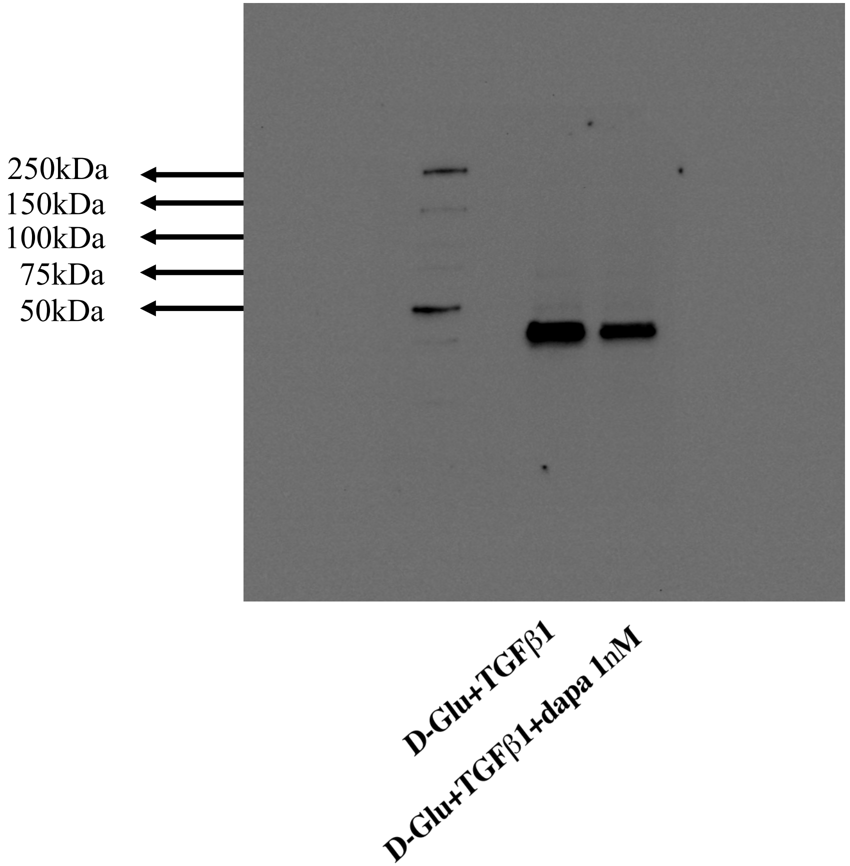

Figure 4C
uncropped blot

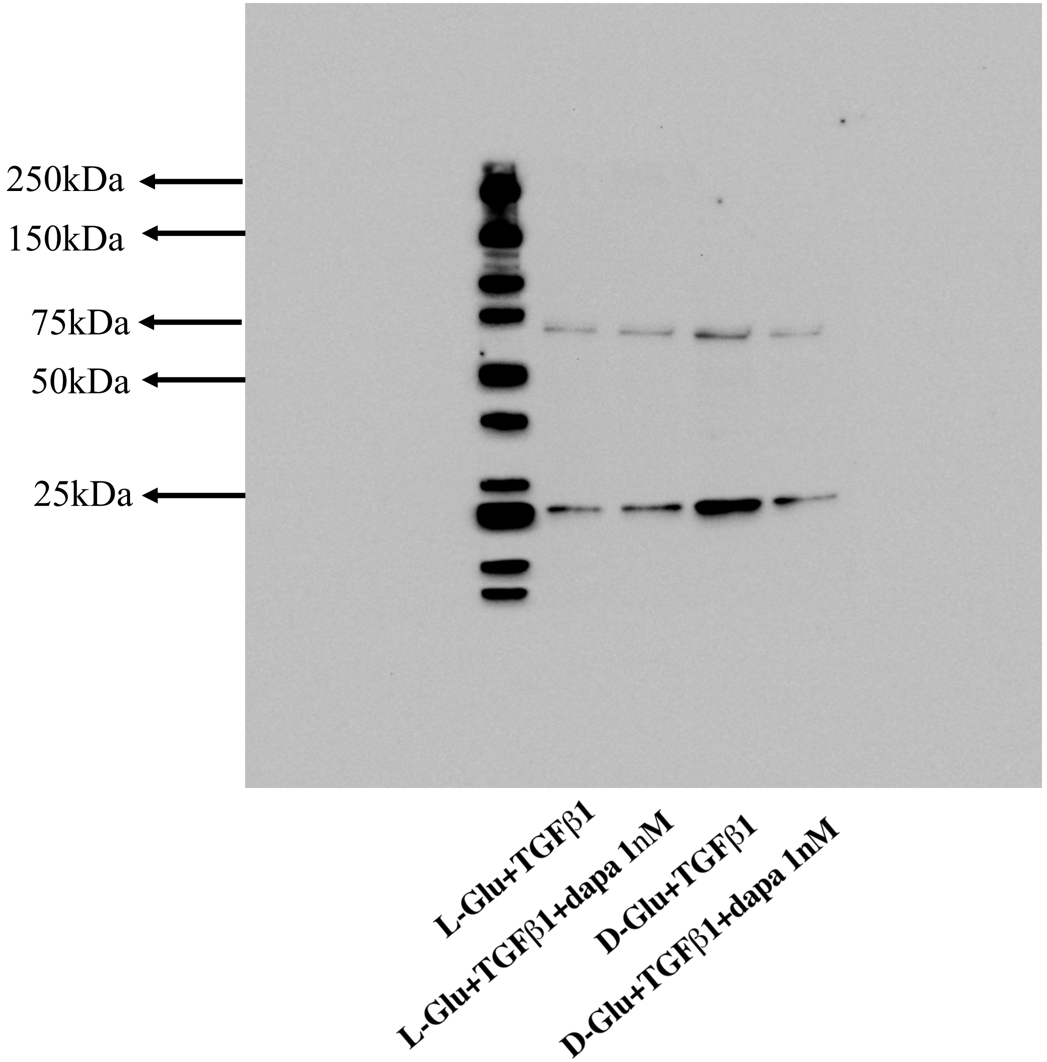

Figure 4E
uncropped blot

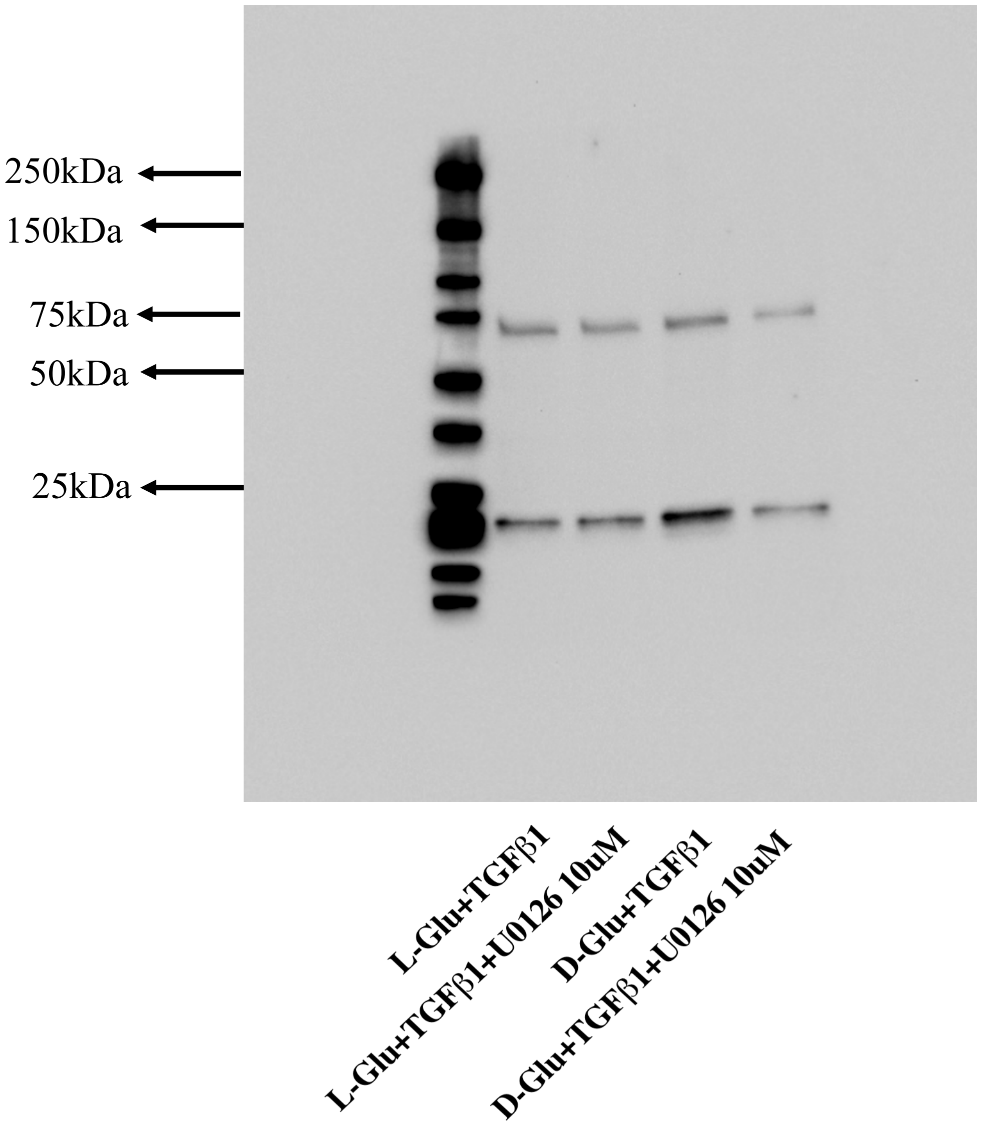

S. Figure 4E uncropped blot

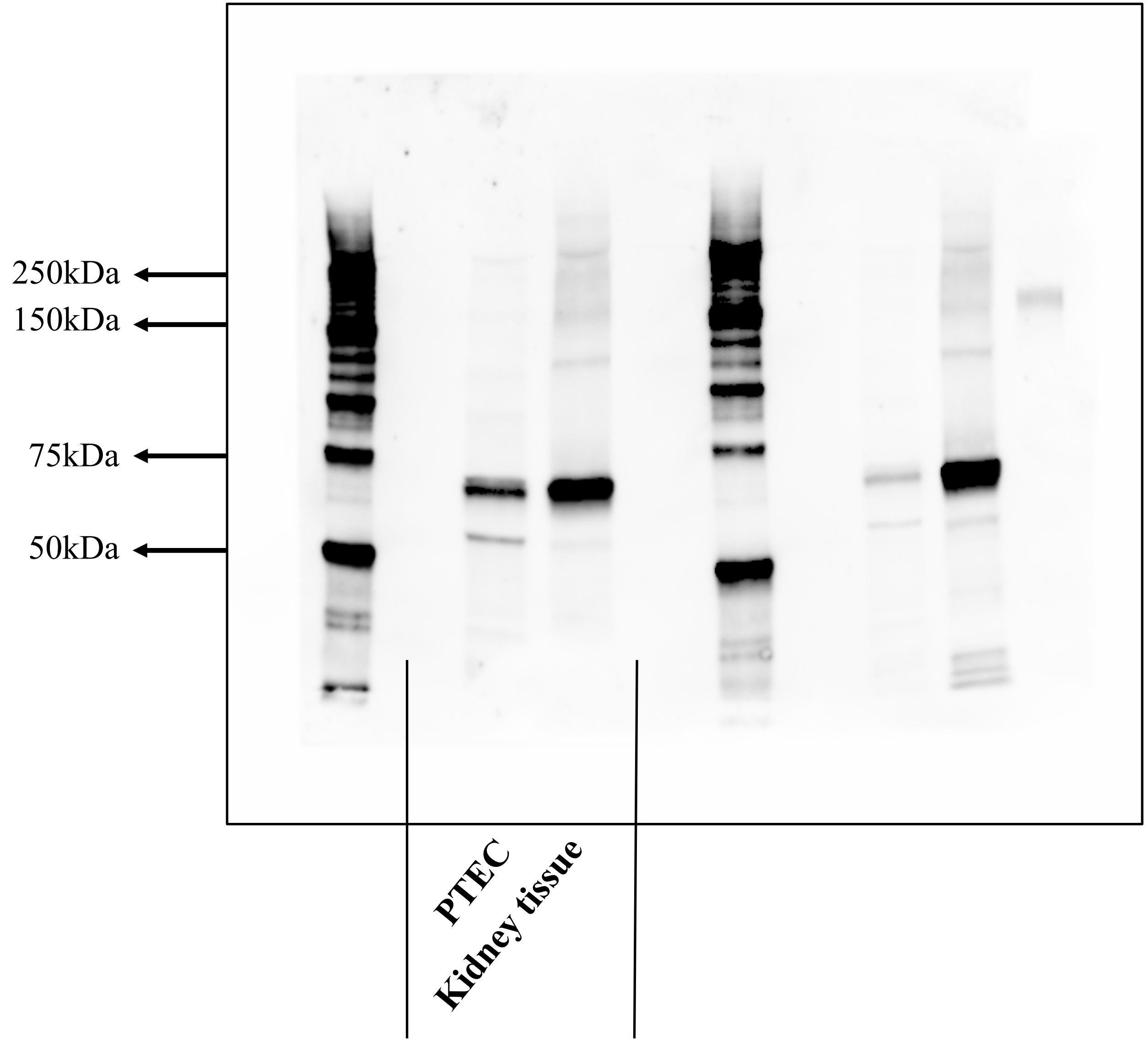

Supplemental Figure 4: Microscopic Image and Gamma GT staining of PTECs

(A) L-Glu and (B) 24 h D-Glu+TGFβ-1 treated PTECs were captured on the microscope (A & B with green filter) to compare any structural changes between the two treatments. (C) L-Glu and (D) D-Glu+TGF-β1 treated cells were also stained with gamma GT to check for positive brush border membrane activity. Distinct orange-red staining is clear in both treatments (E) The SGLT2 antibody used was able to detect an immunoreactive band at the expected weights in both cell lysate and tissue.
